# Supplementary material for: An Immune-Responsive Cytoskeletal-Plasma Membrane Feedback Loop in Plants
Source: Curr Biol. 2018 Jul 9;28(13):2136–2144.e7. doi: 10.1016/j.cub.2018.05.014 (PMC6041470; doi:10.1016/j.cub.2018.05.014)
Supplement: Document S1. Figures S1–S4 and Table S1 [file mmc1.pdf]

Current Biology, Volume *28*

Supplemental Information

An Immune-Responsive Cytoskeletal-Plasma Membrane Feedback Loop in Plants

Stefan Sassmann, Cecilia Rodrigues, Stephen W. Milne, Anja Nenninger, Ellen Allwood, George R. Littlejohn, Nicholas J. Talbot, Christian Soeller, Brendan Davies, Patrick J. Hussey, and Michael J. Deeks

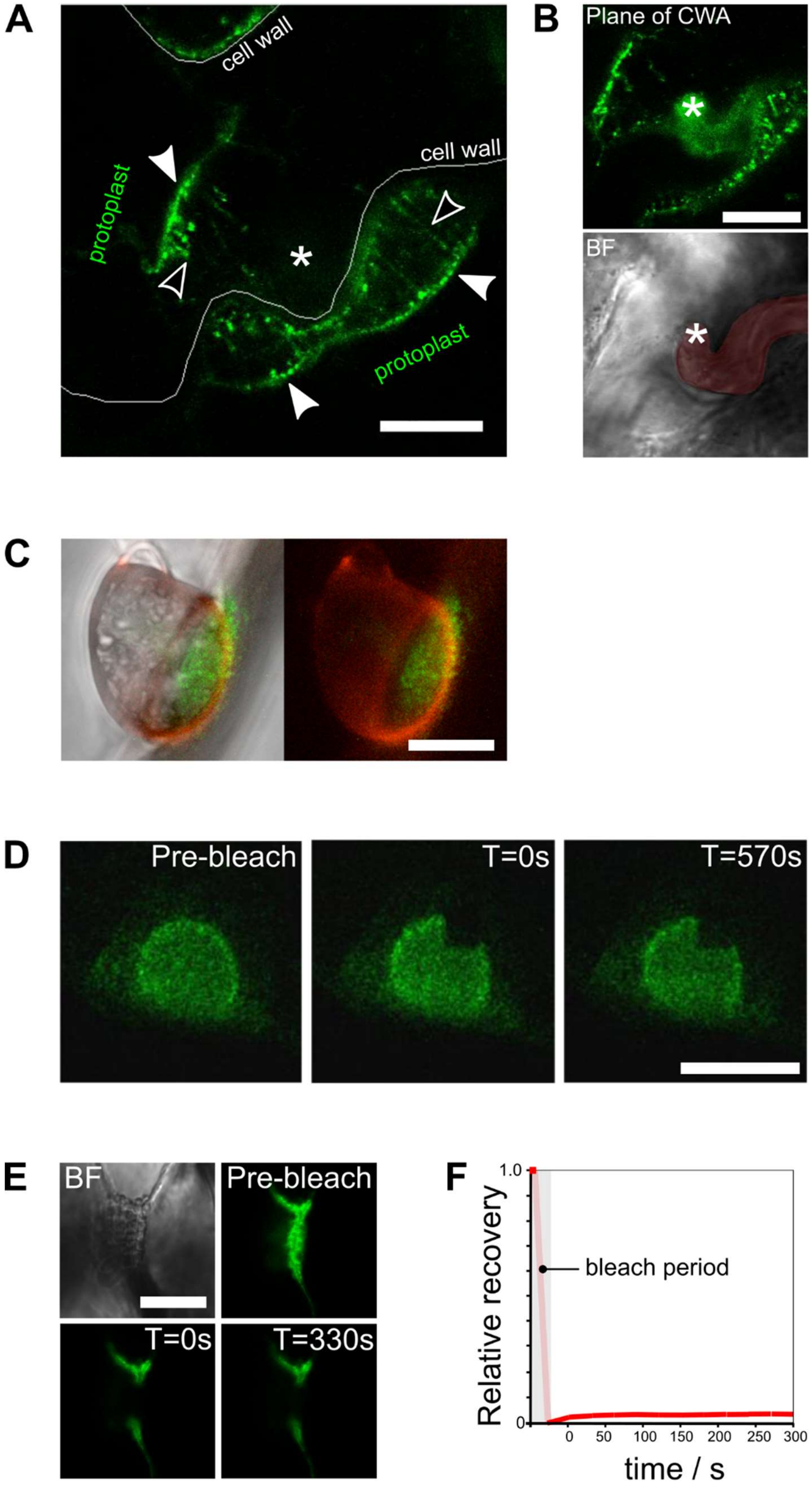

Figure S1. FORMIN4 accumulates in stable deposits at the plant plasma membrane in response to fungi, Related to Figure 1 and Video S1.

(A) Single optical sections of the plasmolysis of *Bgh*-infected epidermal cells expressing FORMIN4-GFP demonstrates that FORMIN4-GFP is associated with the plasma membrane rather than the apoplast. GFP fluorescence can be seen associated with the boundary of the shrinking protoplast. Enriched zones of FORMIN4-GFP membrane are marked with white arrowheads. Hechtian strands of membrane stretching from the cell wall to the protoplast labelled with FORMIN4-GFP are marked with black-filled arrow heads. The asterisk denotes the position of the CWA in the higher imaging plane. Scale bar is 10 µm.

(B) Image plane of the same cell containing the CWA. Asterisk indicates the position of the CWA. Scale bar is 10 µm.

(C) *A. thaliana* epidermal cell expressing FORMIN4-GFP (green channel) in response to a *Magnaporthe oryza* appressorium. Autofluorescence generated by the fungal cell wall can be seen in the red channel. Scale bar is 5 µm.

(D) FORMIN4-GFP discs induced by *Bgh* do not recover from the edges of the bleach zone after photobleaching. Scale bar is 10 µm.

(E) FORMIN4-GFP surrounding *Bgh*-induced CWAs in perpendicular cell walls were used to monitor the mobile GFP fraction during FRAP experiments. Scale bar is 10 µm.

(F) Typical example recovery curve taken from the CWA shown in (B), recording a negligible mobile fraction amounting to less than 5% of the original fluorescence.

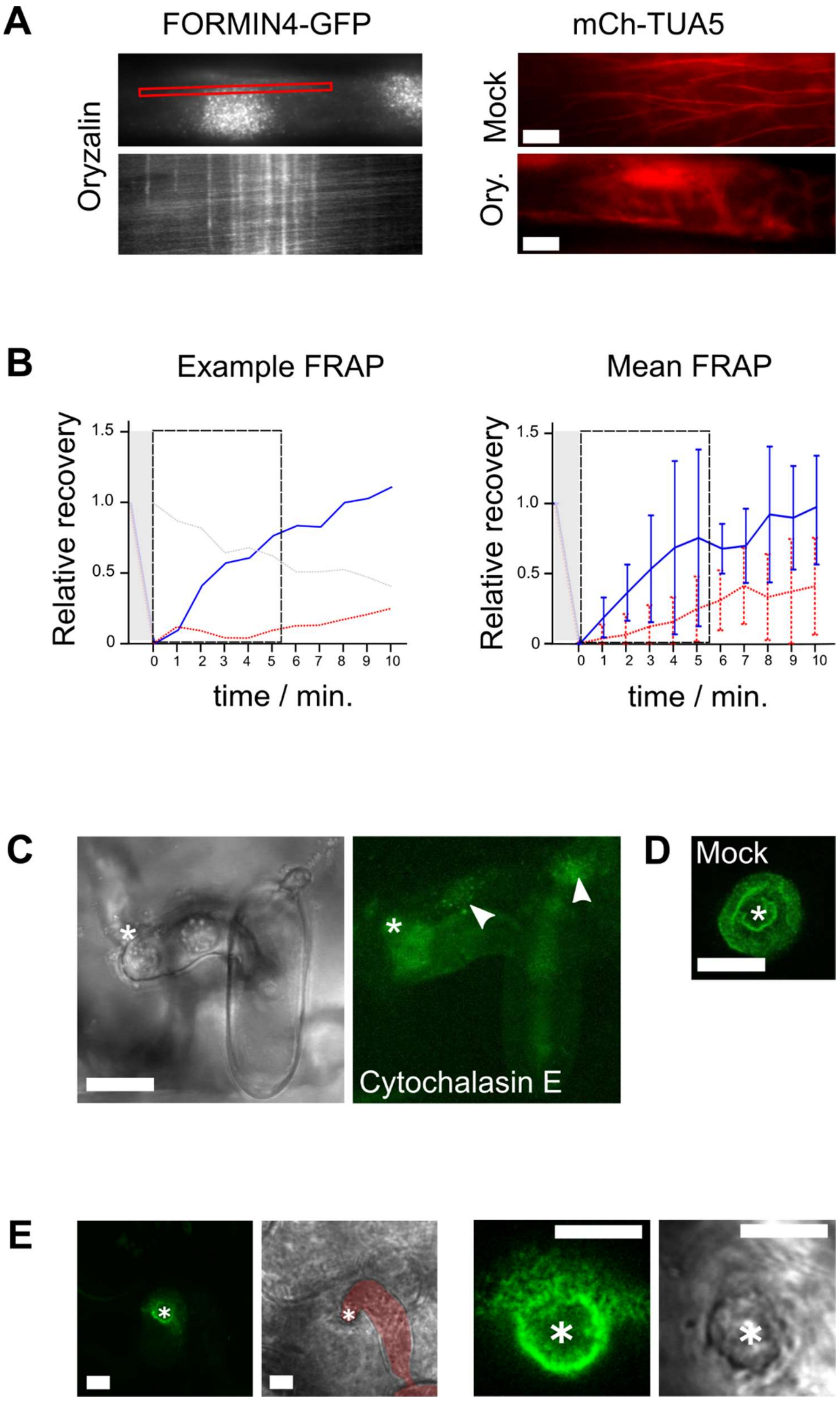

Figure S2. FORMIN4 transport and delivery requires actin but not its endogenous FH1-FH2 domain, Related to Figure 2.

(A) Oryzalin does not disrupt localisation or streaming of FORMIN4-GFP (white). Disruption of microtubules labelled using mCherry-TUA5 (red) confirmed the effectiveness of the oryzalin treatment. Red box is the source of the kymograph in the second panel and is 28.5 µm. Scale bar is 5 µm.

(B) Recovery plots of FRAP data shown in Figure 2. The left-hand plot shows an example recovery of a single FORMIN4-GFP region in an elicitor only (blue line) and latrunculin B (red line) treated hypocotyl. Grey dashed line indicates the reduction of signal in the whole field of view throughout the observation period. Bleach corrections were therefore applied to each data series using control FORMIN4-GFP regions (please see materials and methods). The right-hand plot shows the mean recovery of elicitor only (blue line) and latrunculin B (red line) treated hypocotyls. Black dashed rectangle indicates evaluated time frame for calculating the rates of initial recovery. Error bars are standard deviation.

(C) Infiltration of actin filament capping drug cytochalasin-E prior to *Bgh* infection prevents FORMIN4-GFP accumulation at CWAs. White arrowheads indicate residual responses to fungal contact. Asterisk denotes CWA. Scale bar is 10 µm.

(D) Mock treatments using equivalent concentrations of DMSO do not compromise FORMIN4-GFP delivery. Asterisk denotes CWA.

(E) Two laser scanning confocal microscopy images of *formin4/7/8* expressing FORMIN4-GFP and infected with *Bgh* showing localisation and punctate distribution equivalent to Col-0 genetic background (see Figure 1). Scale bars are 5 µm.

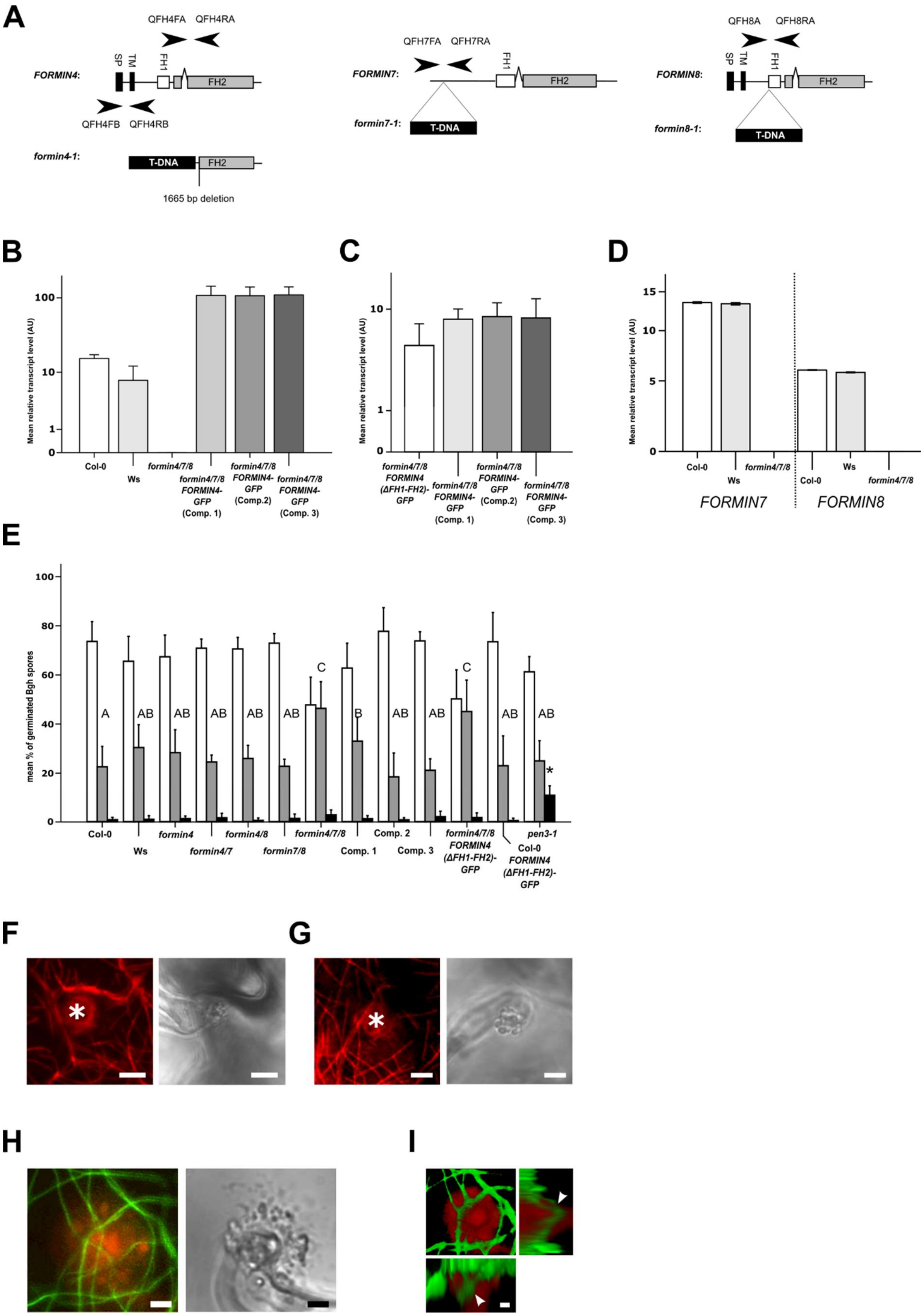
A
QFH4FA
QFH4RA
SP
TM
FH1
FORMIN4:
FH2
QFH4FB
QFH4RB
formin4-1:
T-DNA
FH2
1665 bp deletion
QFH7FA
QFH7RA
FH1
FORMIN7:
FH2
formin7-1:
T-DNA
QFH8A
QFH8RA
SP
TM
FH1
FORMIN8:
FH2
formin8-1:
T-DNA
B
Mean relative transcript level (AU)
100
10
1
0
Col-0
Ws
formin4/7/8
formin4/7/8 FORMIN4-GFP (Comp. 1)
formin4/7/8 FORMIN4-GFP (Comp.2)
formin4/7/8 FORMIN4-GFP (Comp. 3)
C
Mean relative transcript level (AU)
10
1
0
formin4/7/8 FORMIN4 (ΔFH1-FH2)-GFP
formin4/7/8 FORMIN4-GFP (Comp. 1)
formin4/7/8 FORMIN4-GFP (Comp.2)
formin4/7/8 FORMIN4-GFP (Comp. 3)
D
Mean relative transcript level (AU)
15
10
5
0
Col-0
Ws
formin4/7/8
Col-0
Ws
formin4/7/8
FORMIN7
FORMIN8
E
mean % of germinated Bgh spores
100
80
60
40
20
0
A
AB
AB
AB
AB
AB
C
B
AB
AB
C
AB
AB
*
Col-0
Ws
formin4
formin4/7
formin4/8
formin7/8
formin4/7/8
Comp. 1
Comp. 2
Comp. 3
formin4/7/8 FORMIN4 (ΔFH1-FH2)-GFP
Col-0 FORMIN4 (ΔFH1-FH2)-GFP
pen3-1
F
*
G
*
H
I

Figure S3. FORMIN4 contributes to defence against *Bgh*, Related to Figure 3 and Video S2.

(A) Diagram showing the relative positions of T-DNA insertions into mutant group 1e formin alleles. The arrows show the relative position of primers used for RT-QPCR analysis.

(B) Relative expression levels of *FORMIN4* in wild type, mutant and complemented mutant genotypes using primer pair QFH4FA/QFH4RA and control primer pair PTB1F/PTB1R (to housekeeping gene *ptb1*).

(C) Relative expression levels of *FORMIN4* in complemented mutant genotypes using terminal 5-prime primer pair QFH4FB/QFH4RB and control primer pair PTB1F/PTB1R.

(D) Relative expression levels of *FORMIN7 and FORMIN8* in wild type and triple mutant genotypes using primer pairs QFH7FA/QFH7RA and QFH8FA/QFH8RA respectively, in combination with control primer pair EF1F/EF1R (to elongation factor one alpha).

(E) Comparison of the frequency of defence response outcomes in different genotypes 48 hours after *Bgh* infection. These data are from additional biological repeats to those presented in Figure 3. Genotype 'comp.1' is equivalent to the complemented line quantified in Figure 3D. White bars = successful defence/papilla formation, grey bars = response resulted in cell death, black bars = successful haustoria formation. Significant groups were evaluated and defined by a generalized linear mixed model using Tukey HSD as post hoc test. Letters ('A' to 'C') denote groups of no significant difference ($p>0.05$) in proportion of cell death (relative to wild type and triple mutant genotypes). Compared to all other genetic backgrounds *formin4/7/8* and *formin4/7/8 FORMIN4(ΔFH1-FH2)-GFP* plants show increased rates of cell death (group C). Single asterisks indicates a significant difference in haustoria formation of the *pen3-1* mutant to all other tested lines (p-value < 0.001). Of the 129 haustoria recorded 20.2% were alive with no apparent significant difference between genotypes. Error bars show standard deviation of at least three biological repeats with a minimum of 4 leaves (reaching a total of 7119 penetration attempts).

(F) Col-0 leaf epidermal cell 48 hours after *Bgh* infection expressing mCherry-TUA5.

(G) Mutant *formin4/7//8* under the same conditions. Neither wild type nor mutant show microtubule bundling at CWAs. Scale bar is 5 µm.

(H) Maximum projection and accompanying bright field image of a laser scanning confocal microscope image stack of microtubule binding MAP4-GFP (green) and CWA autofluorecence (red) in a wild type genetic background showing microtubules in the zone of the CWA. Arrows indicate a microtubule bundle navigating the invaginated membrane around the CWA. Also see Video S2.

(I) 3D volume rendered image and orthogonal views of the *Bgh* interaction site. Arrowheads indicate microtubules (green) underlying/warping around the papilla (red). Scale bars are 2 µm.

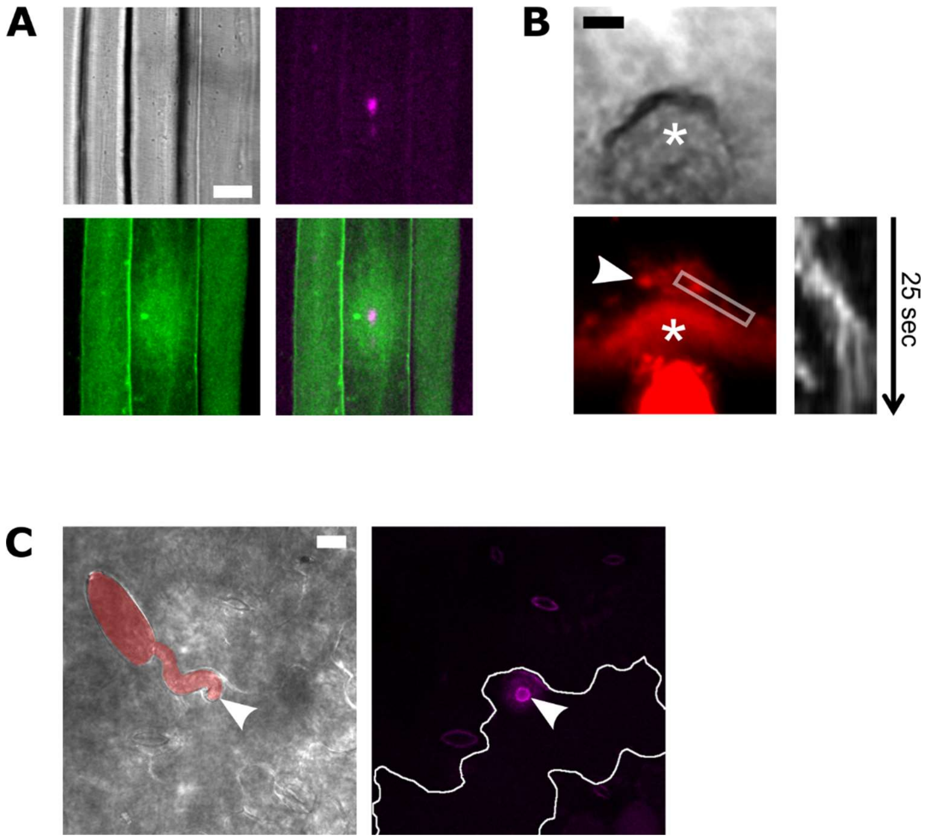

Figure S4. FORMIN4 transport and meso-localisation is distinct from PEN3, Related to Figure 4.

(A) PEN3-GFP (green channel) complementing *pen3-1* dark grown hypocotyls exposed to the elicitation treatment shown in Figure 2. Magenta channel shows aniline blue staining. Scale bar is 10 μm.

(B) The endosomal network (EN; indicated by white arrowhead) surrounding CWAs (example indicated by asterisk) was visualised through the uptake of FM 4-64 (red). Kymograph of the boxed area shows the movement and pausing behaviour of EN compartments. Scale bar is 2 μm.

(C) Tissue infected with *Bgh* expressing FORMIN4-tdTomato. Arrowheads indicate the CWA. Scale bar is 10 μm.

Table S1. Primer combinations for testing of homozygous T-DNA insertion lines.

Related to STAR Methods – Plant material.

Allele	Primer 1	Primer 2
FORMIN4 wild type	FH4GENFA	FH4GENRA
formin4-1 insert	FH4GENRB	TAG3
FORMIN7 wild type	FH7GENFA	FH7GENRA
formin7-1 insert	FH7GENFA	LB3
FORMIN8 wild type	FH8GENFA	FH8GENRA
formin8-1 insert	FH8GENFA	LB3
