# Supplementary material for: An Immune-Responsive Cytoskeletal-Plasma Membrane Feedback Loop in Plants
Source: Curr Biol. 2018 Jul 9;28(13):2136–2144.e7. doi: 10.1016/j.cub.2018.05.014 (PMC6041470; doi:10.1016/j.cub.2018.05.014)
Supplement: Document S2. Article plus Supplemental Information [file mmc5.pdf]

Current Biology

Report

An Immune-Responsive Cytoskeletal-Plasma Membrane Feedback Loop in Plants

Graphical Abstract

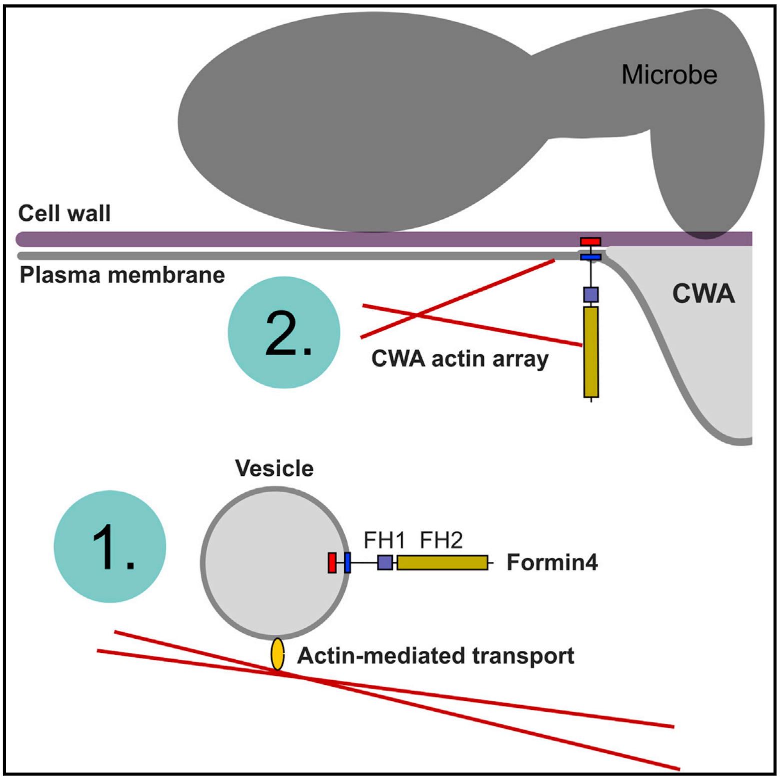

Highlights

- FORMIN4 is delivered to sites of cell wall reinforcement during immune responses
- Delivery requires actin filaments and causes local enhancement of actin dynamics
- Genetic disruption of *FORMIN4* and close homologs causes a defense phenotype
- Transport and localization of FORMIN4 is distinct from key defense protein PEN3

Authors

Stefan Sassmann, Cecilia Rodrigues, Stephen W. Milne, ..., Brendan Davies, Patrick J. Hussey, Michael J. Deeks

Correspondence

p.j.hussey@durham.ac.uk (P.J.H.), m.deeks@exeter.ac.uk (M.J.D.)

In Brief

Sassmann et al. show that the trafficking of host proteins to discrete sites of fungal pathogen contact includes a specific membrane-integrated formin that reinforces local cytoskeletal dynamics during an immune response.

Sassmann et al., 2018, Current Biology *28*, 2136–2144
July 9, 2018 © 2018 The Authors. Published by Elsevier Ltd.
https://doi.org/10.1016/j.cub.2018.05.014

CellPress

Current Biology
Report

An Immune-Responsive Cytoskeletal-Plasma Membrane Feedback Loop in Plants

Stefan Sassmann,[1] Cecilia Rodrigues,[1] Stephen W. Milne,[1] Anja Nenninger,[1] Ellen Allwood,[2] George R. Littlejohn,[1] Nicholas J. Talbot,[1] Christian Soeller,[3] Brendan Davies,[4] Patrick J. Hussey,[2,*] and Michael J. Deeks[1,2,5,*]
[1]Biosciences, University of Exeter, Stocker Road, Exeter EX4 4QD, UK
[2]Department of Biosciences, Durham University, South Road, Durham DH1 3LE, UK
[3]Physics and Astronomy, University of Exeter, Stocker Road, Exeter EX4 4QL, UK
[4]School of Biology, University of Leeds, Miall Building, Leeds LS2 9JT, UK
[5]Lead Contact
*Correspondence: p.j.hussey@durham.ac.uk (P.J.H.), m.deeks@exeter.ac.uk (M.J.D.)
https://doi.org/10.1016/j.cub.2018.05.014

SUMMARY

Cell wall appositions (CWAs) are produced reactively by the plant immune system to arrest microbial invasion through the local inversion of plant cell growth. This process requires the controlled invagination of the plasma membrane (PM) in coordination with the export of barrier material to the volume between the plant PM and cell wall. Plant actin dynamics are essential to this response, but it remains unclear how exocytosis and the cytoskeleton are linked in space and time to form functional CWAs. Here, we show that actin-dependent trafficking to immune response sites of *Arabidopsis thaliana* delivers membrane-integrated FORMIN4, which in turn contributes to local cytoskeletal dynamics. Total internal reflection fluorescence (TIRF) microscopy combined with controlled induction of FORMIN4-GFP expression reveals a dynamic population of vesicular bodies that accumulate to form clusters at the PM through an actin-dependent process. Deactivation of *FORMIN4* and its close homologs partially compromises subsequent defense and alters filamentous actin (F-actin) distribution at mature CWAs. The localization of FORMIN4 is stable and segregated from the dynamic traffic of the endosomal network. Moreover, the tessellation of FORMIN4 at the PM with meso-domains of PEN3 reveals a fine spatial segregation of destinations for actin-dependent immunity cargo. Together, our data suggest a model where FORMIN4 is a spatial feedback element in a multi-layered, temporally defined sequence of cytoskeletal response. This positional feedback makes a significant contribution to the distribution of actin filaments at the dynamic CWA boundary and to the outcomes of pre-invasion defense.

RESULTS AND DISCUSSION

The plant actin cytoskeleton is critical for immune responses to fungi [1, 2] and bacteria [3] and is responsive to pathogenic oomycetes [4, 5]. The impact of actin-mediated trafficking is apparent during hyphal invasion, where the rapid successful assembly of a focused cell wall apposition (CWA) beneath the microbial appressorium and between the plant PM and cell wall prevents hyphal invasion and biotrophic haustorium formation [6]. Molecular, genetic, or chemical interference of actin dynamics during this process lowers penetration defense [1, 2, 7]. To gain mechanistic insight into sustained actin-mediated trafficking in response to microbial interactions, we searched for *A. thaliana* genes encoding known and predicted actin-binding proteins (ABPs). We compared the frequency of transcriptional upregulation (minimal $\log_2$ value of 1) across publicly available transcriptomic experiments, measuring the impact of microbial infection. Of 93 candidates, the most frequently upregulated gene responsive to prokaryote, oomycete, and fungal stimuli (totaling 45 pathogen challenge experiments) encoded *FORMIN4* [8, 9]. We also found this gene was one of only three *ABP* genes present within an established immunity expression cluster [10] (Data S1). Formins are a diverse family of eukaryote cytoskeletal-interacting proteins. Common to most characterized formins are the abilities to stimulate actin nucleation and barbed-end capping through the combined activity of conserved formin-homology 1 (FH1) and formin-homology 2 (FH2) domains (recently reviewed by Shekhar et al. [11]). Many members of the family have been found to have additional capabilities, including actin filament side-binding activity [12], actin filament severing activity [13], and affinity for microtubules [8]. *A. thaliana* FORMIN4 is a member of a unique plant-specific phylogenetic sub-family (plant group 1) that combines an N-terminal secretion signal peptide and transmembrane domain with FH1-FH2 domains within the C terminus [14, 15]. This domain combination has the potential to act as an intimate link between trafficking activity and the cytoskeleton, leading us to consider FORMIN4 a strong candidate for further study.

We confirmed phytopathogen-responsive transcriptional behavior of the *FORMIN4* gene by linking its promoter to the *uidA* reporter gene and infecting stable transformant *A. thaliana* with the powdery mildew *Blumeria graminis* f. sp. *hordei* (*Bgh*) (Figure 1A). *Bgh* is adapted to barley and provokes a non-host response in *A. thaliana* that is commonly used to identify genes contributing to penetration resistance and pre-invasion defense [16, 17]. Next, we made a translational fusion of the complete *FORMIN4* gene, under the control of its own

 © 2018 The Authors. Published by Elsevier Ltd.
This is an open access article under the CC BY license (http://creativecommons.org/licenses/by/4.0/).

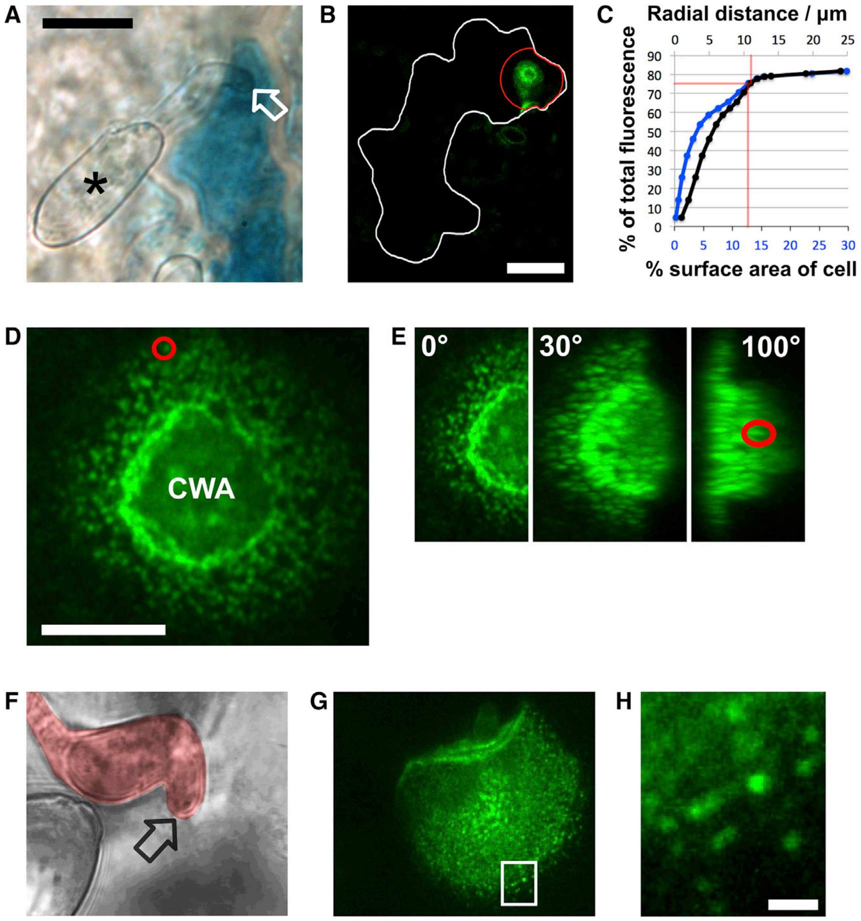

Figure 1. *FORMIN4* Transcript Accumulation Is Activated by Fungi and Localizes to Sites of Fungal Contact

(A) Transcript accumulation of *uidA*-encoded beta-glucuronidase under the control of the *FORMIN4* promoter increases in leaf epidermal cells (as shown by the production of blue precipitate) upon contact with *Blumeria graminis* f. sp. *hordei* (*Bgh*) germ tubes, 48 hr post-infection. Asterisk indicates the *Bgh* spore (conidium), and the white arrow indicates the site of the *Bgh* penetration peg (appressorium). The scale bar represents 20 μm.

(B) A translational fusion of GFP to *FORMIN4* localizes specifically to the site of interaction. White line shows the boundary of the epidermal cell contacted by *Bgh* (48 hr post-infection), and red line shows the radial distance from the fungus containing 80% of the GFP signal (the site of fungal contact at the center of the red zone was determined using a transmission image). The scale bar represents 20 μm. See also Figure S1.

(C) Graph showing proportion of fluorescence versus radial distance (black line) and cell surface area (blue line) from the point of hyphal contact for the cell in (B).

(D) High-resolution imaging of the plant plasma membrane at the contact site shows that GFP is segregated into punctate domains of approximately 200 nm diameter. The red circle highlights an example puncta. "CWA" indicates the location of the cell wall apposition. The scale bar represents 5 μm.

(E) Three-dimensional projections show that the punctate pattern is maintained at the plasma membrane (example puncta indicated by red circle) surrounding the CWA. See also Figure S1 and Video S1.

(F–H) *Bgh* appressorial germ tube contact sites (indicated by black arrow; F) can be identified with the same punctate pattern (G and H) at 16 hr post-infection, without penetration peg ingression. The scale bar represents 1 μm.

promoter, to GFP and imaged stable *A. thaliana* transformants infected with *Bgh*. GFP fluorescence was detected specifically in epidermal cells in contact with fungal structures (Figure 1B), further confirming the transcriptomic analysis and the reporter gene experiments. Moreover, the fusion product was found almost exclusively in the locality of CWAs formed in response to the fungus (Figures 1B and 1C). Induced plasmolysis of infected cells demonstrated that the GFP fluorescence was associated with the host cell plasma membrane (PM) rather than the fungus or the plant cell wall (Figures S1A and S1B). High-resolution imaging revealed that the fluorescence was compartmentalized into small puncta of 184 ± 24 nm in diameter (measured using an Airyscan instrument; Figure 1D) that were retained at the PM during plasmolysis induction (Figures S1A and S1B). This dense punctate pattern continues across the periphery of the CWA as the PM wraps around the material deposited below the plant cell wall (Figure 1E; Video S1). Samples observed between 16 and 24 hr post-infection showed examples of FORMIN4-GFP accumulation in response to *Bgh* primary and appressorial germ tubes without differentiation or ingression of a penetration peg (Figures 1F–1H). FORMIN4-GFP accumulation can therefore occur without full CWA development (Figures 1F and 1G). This is a behavior shared by other membrane-integrated proteins trafficked during immune responses (such as PEN3) and suggests such trafficking can be guided by molecules released at the site of pathogen contact [18].

Recruitment of proteins to the local PM by pathogens can be either part of a basal defense response or caused by the action of a specific fungal infection mechanism. To differentiate between these two possible scenarios, we infected FORMIN4-GFP stable transformants with *Magnaporthe oryzae*, as this fungal phytopathogen has a distinct infection strategy [19, 20] and does not exploit the same host mechanisms required for *Bgh* infection [21]. FORMIN4-GFP was observed to localize to the PM below differentiated *M. oryzae* appressoria (Figure S1C). These data suggest that FORMIN4-GFP transport occurs as a broad-spectrum fungal-response mechanism.

We next developed an assay to observe FORMIN4-GFP dynamics at early stages post-stimulation using total internal reflection fluorescence (TIRF) microscopy. This would enable probing of the mechanism of FORMIN4 delivery, as mature CWAs imaged using confocal microscopy did not show quantifiable delivery dynamics (Figures S1D–S1F). Contacts between

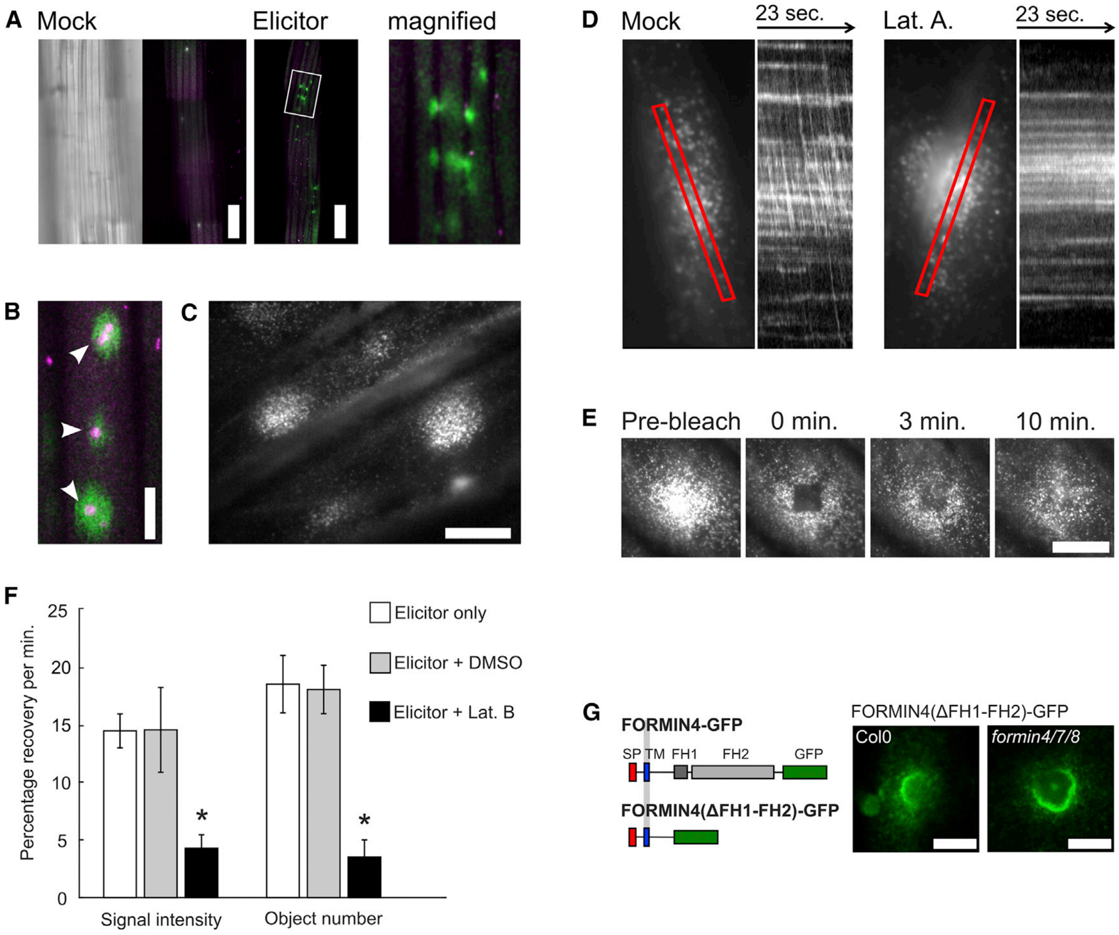

Figure 2. FORMIN4 Transport and Delivery Require Actin, but Not Its Endogenous FH1-FH2 Domain

(A) Mosaics consisting of multiple confocal laser scanning microscope images combined to show wide areas of dark grown FORMIN4-GFP transformant hypocotyls. Left: the corresponding transmission image to the mock-treated sample. Right: FORMIN4-GFP signal after 4 hr elicitation. Cells expressing FORMIN4-GFP after elicitation showed an average number of 1.98 (±1.16) FORMIN4-GFP regions per GFP-positive cell (n = 64). The scale bars represent 100 μm.

(B) Aniline blue (magenta) staining shows that the majority of FORMIN4-GFP regions are associated with centralized callose regions (88.9% ± 8.6%). The scale bar represents 10 μm.

(C) Typical TIRF microscopy image of regional FORMIN4-GFP accumulation after 4 hr of elicitation. The scale bar represents 10 μm.

(D) FORMIN4-GFP expression is induced and delivery achieved four hours after immune system stimulation. Kymographs (taken along the lengths of indicated red boxes) show that FORMIN4-GFP-labeled vesicular bodies can be detected in the cytoplasmic stream. Latrunculin A and B treatment disrupts streaming, but plasma membrane-associated FORMIN4-GFP remains in a stable pattern. Red boxes are 25 μm in length. See also Figure S2.

(E) Fluorescence recovery after photobleaching (FRAP) experiments performed using total internal reflection fluorescence (TIRF) microscopy demonstrate that the site-specific delivery process is active four hours after stimulation. See also Figure S2. The scale bar represents 10 μm.

(F) The delivery process is interrupted by latrunculin B treatment as measured using pixel fluorescence recovery and recovery of object number. Asterisk indicates a significant difference of $p < 0.05$. Error bars indicate the SE. See also Figure S2.

(G) Removal of the FORMIN4 cytosolic domain (that includes the FH1-FH2 domain) does not prevent FORMIN4 localization to the infection site either in wild-type plants or in plants where all group 1e formins have been genetically disrupted. The scale bar represents 5 μm.

hyphae and plant cells are asynchronous, and appressoria obstruct PM imaging. To overcome these technical challenges, we used an elicitor to approximate the molecular patterns of fungal assault. This consisted of a dilute plant cell wall hydrolyzing enzyme mix supplemented with chitin and endochitinase to generate chitin oligomers. Increased GFP fluorescence was observed after *A. thaliana* tissue was incubated for a minimal period of four hours in the elicitation mix (Figure 2A). This further confirms the FORMIN4 response is not species specific to *Bgh* and can be induced in the absence of specific fungal–disease promoting effectors. FORMIN4-GFP was concentrated at the PM in bright, dense regions of varying diameter (9.5 ± 2.6 μm) with multiple regions often found in a single cell (on average 2.0 ± 1.2 per cell). Transmission images of these regions frequently suggested the presence of local cell wall aberrations. We stained FORMIN4-GFP hypocotyl cells with aniline blue to highlight deposits of callose, an injury and immune-responsive cell wall polymer and a major component of CWAs. We found that 88.9% (±8.6%) of FORMIN4-GFP regions coincided with small callose deposits (Figure 2B). The localized regions of FORMIN4-GFP are therefore associated with sites of cell wall reinforcement.

At higher magnification and resolution, the FORMIN4-GFP distribution resembled the punctate pattern observed at the PM surrounding CWAs in *Bgh*-infected tissue (Figure 2C). A population of rapidly moving vesicular bodies could be detected in areas of the cytoplasmic stream close to the PM with vesicular structures visibly exchanged at stream-PM contact sites (Figure 2D). This movement is sensitive to chemical disruption of the actin cytoskeleton (Figure 2D), but not the microtubule cytoskeleton (Figure S2A). Neither class of treatment caused disassociation of FORMIN4-GFP puncta from the PM.

We used a fluorescence recovery after photobleaching (FRAP) approach combined with TIRF microscopy to test the hypothesis that actin-mediated motility plays a role in the arrival of FORMIN4 content. 25 μm^2 areas of concentrated FORMIN4-GFP puncta were bleached and fluorescence recovery monitored over the subsequent 10 min, during which time the puncta content increased (at a relative recovery rate of 14.4% ± 4.9% per minute; Figures 2E, 2F, and S2B). FRAP experiments were also performed after acute exposure to latrunculin B and mock treatments. The mock treatment did not affect the rate of FORMIN4-GFP content increase whereas latrunculin B significantly reduced the rate to 34% of control levels (Figure 2F). This suggested that the actin cytoskeleton is critical for the delivery process. To validate these data *in vivo*, we pre-treated *Bgh*-infected *A. thaliana* leaves stably transformed with FORMIN4-GFP with cytochalasin E, a fungus-derived secondary metabolite that compromises the plant actin cytoskeleton but does not affect *Bgh* development [2]. FORMIN4-GFP distribution was severely reduced and poorly targeted (Figures S2C and S2D). Together, these data show that FORMIN4-GFP delivery to immune response sites requires a functioning actin cytoskeleton.

The dependence of FORMIN4-GFP vesicle transport on the actin cytoskeleton raises the hypothesis that the FH1-FH2 domains of FORMIN4 are significantly contributing to vesicle-filament interactions and acting in a *cis* fashion to drive the distribution of vesicles with FORMIN4 surface content. To address this question, we generated a deletion construct that contained only the secretion signal peptide, transmembrane domain, and intervening sequence fused to GFP. This deletion mutant (FORMIN4(ΔFH1-FH2)-GFP) did not contain the FH1 and FH2 domains (Figure 2G). When transformed into wild-type plants, this minimal fragment was delivered to *Bgh* sites (Figure 2G). We generated a triple T-DNA insertion mutant with a disrupted allele of *FORMIN4* and the two closest homologs of *FORMIN4* (*FORMIN7* and *FORMIN8*; constituting the complete phylogenetic group 1e; Figure S3A). Both FORMIN4-GFP (Figure S2E) and FORMIN4(ΔFH1-FH2)-GFP (Figure 2D) fusion proteins were delivered to *Bgh* sites in this genetic background. The actin-interacting potential of FORMIN4 is therefore unlikely to have an essential function in a *cis* capacity during vesicle delivery.

The generation of the *formin4/7/8* mutant provided an opportunity to screen for phytopathogen defense phenotypes and to test the functionality of the FORMIN4-GFP construct. We scored the frequency of different outcomes from *Bgh-A. thaliana* interactions in wild-type, *formin4/7/8*, and *formin4/7/8* plants complemented with FORMIN4-GFP. At forty-eight hours post-infection, individual outcomes of appressorial attack were classified as intact CWAs associated with living cells (Figure 3A), intact CWAs combined with cell death (Figure 3B), and breached CWAs with fungal haustorium development (Figure 3C). The *formin4/7/8* plants showed a significant increase in both the frequency of cell death and a small but significant increase in the frequency of haustoria development (Figure 3D). Expression of FORMIN4-GFP reduced the presence of haustoria and almost completely restored the normal frequency of cell death in the absence of haustoria, reducing the frequency from 48.4% (±6.8%) to 36.0% (±5.0%), close to the wild-type frequency of 29.5% (±6.1%). This demonstrates that FORMIN4-GFP retains sufficient biological activity to complement the function of group 1e formins and that FORMIN4 contributes significantly to a defense response against a fungus. No complementation of the *Bgh*-response phenotype was detected in cells expressing FORMIN4(ΔFH1-FH2)-GFP (Figure S3E).

To better classify the phenotype, we compared the formin triple mutant to a characterized resistance mutant, *pen3-1*. PEN3 is a membrane-integrated ABC transporter protein with a critical role in defense that is enriched at the PM surrounding CWAs in a process dependent upon the actin cytoskeleton [22, 23]. The *pen3-1* mutant allele affects the profile of secondary metabolites transported by PEN3 [24] and compromises defense responses to a broad range of pathogens, including *Bgh* [16]. The *pen3-1* mutant plants showed an elevated level of haustoria formation beyond that of the formin triple mutant; however, the formin triple mutant showed a greater proportion of cells entering cell death after CWA formation (Figure S3E). We therefore conclude that the formin triple mutant causes a subtly different *Bgh* pre-invasion response phenotype to the *pen3-1* exemplar of a penetration mutant.

The actin cytoskeleton is known to support CWA function [1, 2, 25] (e.g., Figure S2C), and formins are known to modify local actin dynamics. We imaged infected *formin4/7/8* plants expressing GFP-Lifeact [26] to test the hypothesis that the actin cytoskeleton had been compromised. This revealed an intact actin cable network broadly comparable to wild-type plants (Figures 3E and 3F). In both genotypes, actin cables exhibited transient interactions with the periphery of the CWA that maintained a local cytoplasmic stream. No equivalent enrichment of microtubules was observed in either wild-type or mutant plants expressing an mCherry fusion to tubulin (mCherry-TUA5; Figures S3F and S3G), an observation that contrasts the cereal interactions with powdery mildew [27] but is in agreement with previously reported non-host responses to *Bgh* in *A. thaliana* [28]. Surprisingly, actin cable interactions with CWAs in the mutant background appeared to be more direct with reduced peri-CWA regions containing finer dispersed F-actin structures (Figures 3G and 3H). In wild-type leaves, 80.4% (±17.4%) of uncompromised CWAs in living cells were associated with a radially symmetrical fringe network of F-actin whereas 16.8% (±7.3%) of mutant CWAs retained similar networks. This phenotypic behavior suggests a contribution of FORMIN4 to the cortical interactions and network properties of PM-associated actin filaments, a role consistent with the biochemical behavior of FORMIN4 [8, 9] and phenotypes of group 1 family members [29, 30]. Together, these data suggest an actin-dependent trafficking-driven mechanism for reinforcing cytoskeletal behavior.

We next asked whether the FORMIN4 transport route and localization pattern was shared with PEN3. The key defense

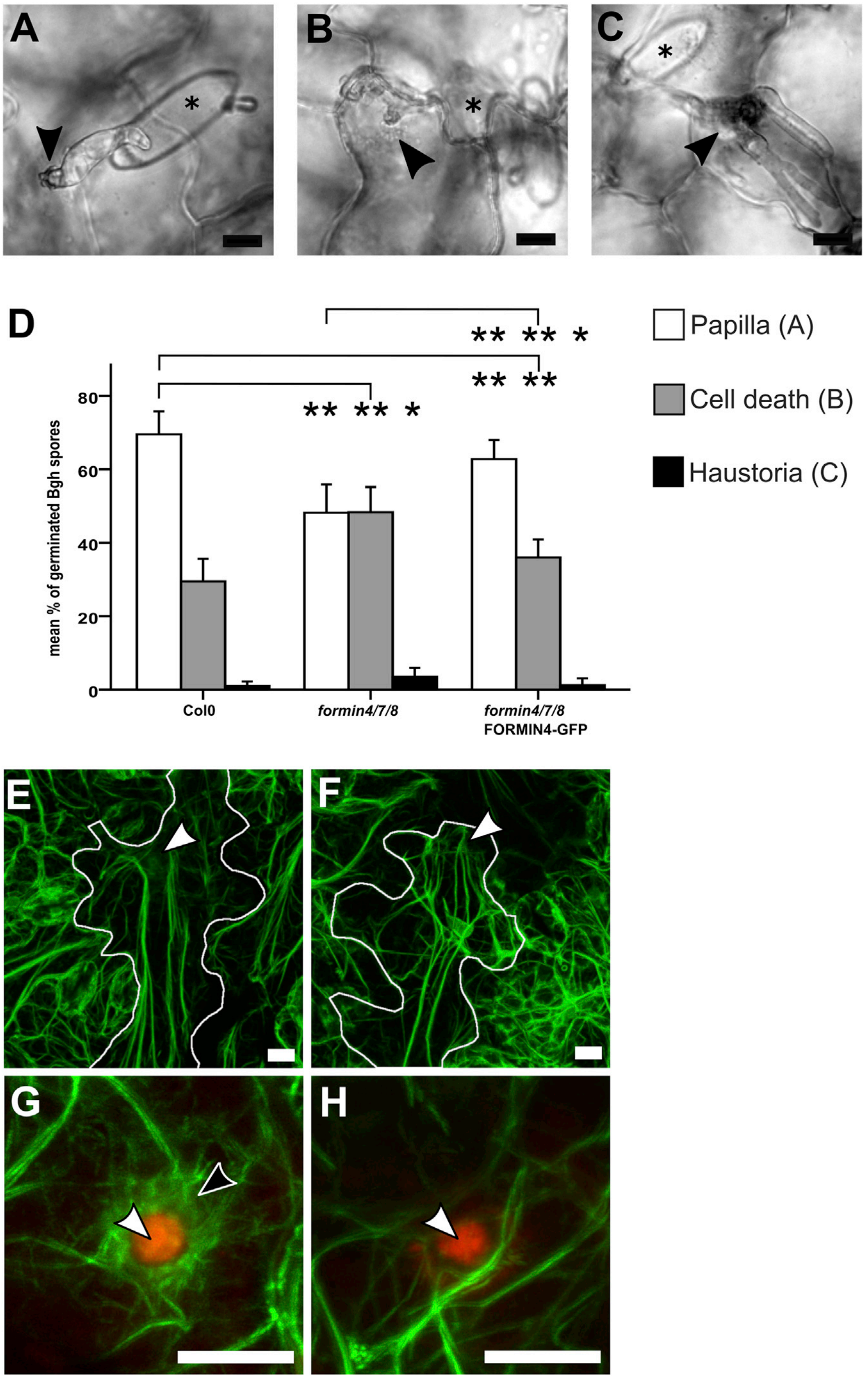

Figure 3. FORMIN4 Contributes to Defense and Actin Organization at the Site of Immunity

(A) At 48 hr after infection, most wild-type *A. thaliana* epidermal cells responding to *Bgh* appressoria have formed a CWA (indicated by arrowhead).

(B) A proportion of cells enter programmed cell death, identified by absence of cytoplasmic streaming, aggregation of the cytoplasm, and pigmentation of the cell.

(C) A small minority of cells contain fungal haustoria. Asterisk in (A)–(C) indicates conidia.

(D) Comparison of the frequency of responses in different genotypes. Compared to wild-type (Col-0), *formin4/7/8* plants show increased rates of cell death and haustoria formation upon *Bgh* challenge. Expression of FORMIN4-GFP in the *formin4/7/8* genetic background fully rescues mutant susceptibility to haustoria formation and reduces cell death rates near to wild-type levels. Single asterisks indicate p values less than 0.05; double asterisks indicate p values less than 0.01 (evaluated by pairwise comparison applying Student's t test). Error bars show SD of at least three biological repeats (minimum of 22 leaves per genotype). See also Figure S3.

(E) Wild-type cells expressing actin-binding GFP-Lifeact show a cable network that interacts with the region containing the CWA (labeled using a white arrowhead; white lines mark the boundary of the affected epidermal cell).

(F) The CWA (position indicated by white arrowhead) maintains interactions with the actin cable network in mutant *formin4/7/8* plants expressing GFP-Lifeact.

(G) GFP-Lifeact-labeled filaments (green) surrounding wild-type CWAs (red autofluorescence and white arrowhead). Black arrowhead indicates coronal (peri-CWA) actin network.

(H) GFP-Lifeact labeling filamentous actin in the area surrounding a mutant CWA.

The scale bars represent 10 μm.

protein PEN3 also accumulates at CWAs and smaller depositions of callose (Figure S4A) in an actin-dependent process. To approach the trafficking question, we used the uptake of the fluorescent lipophilic dye FM4-64 to label the endosomal network (EN) and quantify co-localization with FORMIN4-GFP and PEN3-GFP. PEN3 is found in early endosomal compartments [31] that are mobile on actin filaments, and the EN is engaged in the trafficking of PM-derived multi-vesicular bodies to the CWA interior [32, 33]. A ninety-minute incubation of infected tissue with FM4-64 did not label the vacuole membrane or endoplasmic reticulum but did highlight mobile compartments that showed pausing behavior in the region of CWAs (Figure S4B). We used an object-recognition and centroid-distance-based approach (Figures 4A–4D) to quantify the co-localization of these EN compartments with GFP fusion proteins. 26.6% (±9.5%) of FM4-64-labeled compartments co-localized with PEN3-GFP objects whereas only 5.6% (±3.9%) of these compartments had locations within 200 nm of FORMIN4-GFP objects. Furthermore, the distribution of paired distances supports the existence of a PEN3-GFP peak sub-population co-localizing with FM4-64 but no equivalent FORMIN4-GFP population (Figures 4B–4D). To address the question of co-localization, we used a FORMIN4-tdTomato fusion protein co-expressed with PEN3-GFP to test whether the two proteins occupy the same zone of PM. Both FORMIN4-tdTomato and PEN3-GFP were enriched at the

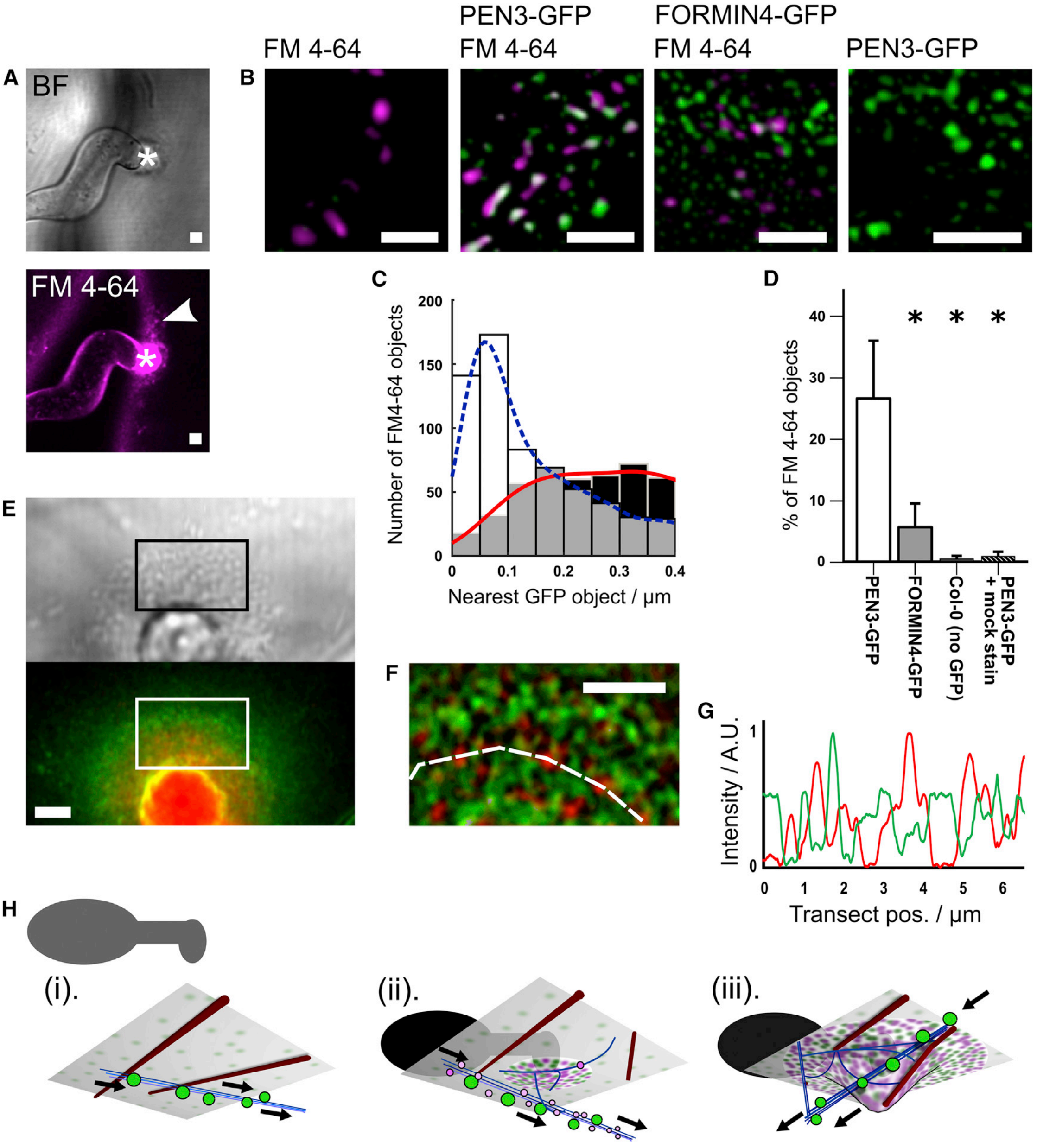

Figure 4. FORMIN4 Transport and Meso-localization Are Distinct from PEN3

(A) Bright field image of *Bgh* appressorial germ tube contact site with CWA (indicated by asterisk) and endosomal network (EN) compartments (indicated by white arrowhead) visualized by FM4-64 uptake (magenta). The scale bars represent 2 µm. See also Figure S4.

(B) Laplacian of Gaussian (LoG) filtered images of simultaneous detection of FM4-64-labeled EN compartments (magenta) and GFP expression (green) in wild-type (Col-0), PEN3-GFP, FORMIN4-GFP, and mock-treated PEN3-GFP cells. Mock treatment of PEN3-GFP resulted in no visible EN vesicles in the FM4-64 detection wavelength, and FM4-64-treated wild-type samples showed no signal above background within the GFP excitation/emission channel. The scale bars represent 2 µm.

(C) Distance-based quantification of co-localization between FM4-64-labeled compartments and GFP-labeled objects shows that a peak sub-population of PEN3-GFP objects, but not FORMIN4-GFP objects, coincide at distances less than the diffraction limit of the microscope. Histogram represents centroid distance of PEN3-GFP compartments to EN compartments (white bars and blue Gaussian kernel regression) and FORMIN4-GFP to EN compartments (black bars and red Gaussian kernel regression). Grey areas show histogram overlap.

(D) PEN3-GFP co-labels 26.6% (±9.5%) of the EN objects, whereas only 5.6% (±3.9%) of the EN is within equivalent distances to FORMIN4-GFP objects. Asterisk indicates a p value evaluated by pairwise comparison with Student's t test of <0.001. Error bars show SD.

(E) Co-expressed FORMIN4-tdTomato (red) and PEN3-GFP (green) at the plasma membrane in the region of a CWA. See also Figure S4. The scale bar represents 2 µm.

(F) Bandpass phase-frequency filtering highlights the local enrichment of FORMIN4-tdTomato and PEN3-GFP plasma membrane domains (area enlarged from white box in D) showing tessellation of FORMIN4 and PEN3 enrichments. The scale bar represents 2 µm.

(legend continued on next page)

PM, with FORMIN4-tdTomato showing the same extreme CWA spatial restriction as FORMIN4-GFP (Figure S4C). Higher magnifications suggested that both proteins were enriched in a punctate pattern at the PM (Figure 4E). Phase-frequency filtering of these data enhances the contrast between the two patterns (Figure 4F), and an arc transect shows that peaks and troughs of the two proteins do not coincide (Figure 4G). We therefore conclude that, although PEN3 and FORMIN4 trafficking are both actin dependent, they show little co-compartmentalization in either the EN or the PM at the meso-scale. Actin-mediated immune-responsive trafficking can therefore be sub-divided into different routes and subtly different destinations. FORMIN4, and any potentially associated PM content, is largely segregated from the endosomal transport network. This could explain its long-term persistence at the PM and the absence of a large mobile fluorescence fraction at fully developed CWAs (Figures S1D–S1F).

Here, we have shown that plant cells respond to the challenge of pathogenic microbes by placing a stable molecular flag at the destination of their secretion pathway. We propose a model (Figure 4H) where pathogen contact stimulates targeted exocytosis of marker cargo, including FORMIN4. This initial activation is likely to occur through the detection of microbial and damage-associated molecular patterns (MAMPs and DAMPs). This is supported by studies of targeted PEN3 transportation [18] and the activation of FORMIN4 delivery through molecular patterns (Figure 2). Actin dynamics can be stimulated by MAMP application [3], with compelling evidence suggesting that this occurs, at least in part, through the actions of ADF and capping protein [3, 34]. To date, the spatial specificity of these mechanisms has not been studied, but targeted FORMIN4 delivery is actin dependent and it seems plausible that transient modification of cytoskeletal dynamics local to the microbial contact site supports any initial burst of trafficking. Logically, alternative systems must be upstream in temporal sequence from actin binding proteins, such as FORMIN4, that are under tight expression control and embedded in the cargo membrane. Long-term accumulation of FORMIN4 (and potentially other factors resistant to uptake into the EN) further reinforces the local actin-filament distribution network. Group 1 formins show little lateral diffusion within the PM (so long as the PM remains in contact with the cell wall) [35, 36] and are therefore highly adapted to the purpose of acting as persistent markers of PM identity with a capability to support local transport networks. The extended "new" surface of the CWA supports stable cytoskeletal interactions (Figures S3F–S3I; Video S2). The peri-CWA actin and microtubule networks can facilitate a variety of activities, including local organelle positioning, vesicular trafficking to and from the plasma membrane, and greater perception of the microbial stimulus. *A. thaliana* retains eleven genes encoding group 1 formin proteins as well as numerous group 2 formins and the Arp2/3 complex. This provides considerable opportunity for functional redundancy with parallel actin-nucleation pathways and is likely to prevent catastrophic collapse of actin filament turnover. Drug-induced actin disruption in *A. thaliana* allows *B. graminis* haustorium formation, but full disease progression is prevented by programmed cell death pathways [25]. The *formin4/7/8* mutant therefore accelerates this defense outcome associated with actin filament deficiency without providing opportunity for haustorium establishment. This could reflect direct monitoring of peri-CWA actin dynamics by the plant immune system or a "tipping of the balance" toward programmed cell death in the face of mildly inefficient CWA formation. Our observation of PEN3/FORMIN4 meso-domain tessellation at this interface provides a potential strategy for achieving FORMIN4 stability while simultaneously providing a site for high-volume delivery to the apoplast. Understanding the molecular basis for these multiple scales of organization will likely reveal new aspects of disease virulence and promises the means to deliver bespoke anti-microbial cargoes with high precision.

STAR★METHODS

Detailed methods are provided in the online version of this paper and include the following:

- KEY RESOURCES TABLE
- CONTACT FOR REAGENT AND RESOURCE SHARING
- EXPERIMENTAL MODEL AND SUBJECT DETAILS
  - Plant material
  - Plant growth conditions
  - Pathogen growth conditions
  - Powdery mildew infection assay – growth conditions
- METHOD DETAILS
  - Powdery mildew infection assay
  - *Magnaporthe oryzae* infection
  - Plasmids
  - GUS Analysis
  - RNA purification and reverse transcriptase
  - RT-qPCR
  - Hypocotyl elicitation and imaging
  - Drug treatments
  - Leaf plasmolysis
  - Aniline blue staining of callose deposits
  - Infected leaf FM4-64 uptake
  - FRAP analysis
  - Analysis of publicly available microarray data
  - Co-localization analysis
- QUANTIFICATION AND STATISTICAL ANALYSIS

(G) Amplitude of transect taken from the dashed line in (E). Peaks of FORMIN4-tdTomato (red) do not coincide with peaks of PEN3-GFP (green).

(H) Model proposing a sequence of events leading to positional feedback during an immune response. The zero time point (i) shows wide PM distribution of PEN3 (green patches) and sub-cortical PEN3-containing EN compartments (green spheres) mobilized on the actin network (blue lines; microtubules are represented by brown rods). Arrowheads indicate the unidirectional flow of the local cytoplasmic stream. Local perception of the appressorium (background silhouette) is about to initiate. By four hours (ii) post-perception, FORMIN4-containing vesicles (magenta spheres) are present within the cytoplasmic stream and content is arriving at the nascent response zone of the PM in an actin-dependent process. By forty-eight hours (iii), a fully developed CWA has intercepted the penetration attempt and has accumulated distinct PM content, including stable FORMIN4. The cytoskeleton accommodates and engages with the new PM topography, in part through the earlier actin-dependent deposition of PM proteins.

SUPPLEMENTAL INFORMATION

Supplemental Information includes four figures, one table, two videos, and one data file and can be found with this article online at https://doi.org/10.1016/j.cub.2018.05.014.

ACKNOWLEDGMENTS

We are extremely grateful to John Steele, Lauren Mortimer, Natalia Fenyk, and Zeiss Microscopy's UK confocal sales and technical team for their technical support during this project. We would also like to thank Frederic Berger, Shauna Somerville, Tijs Ketelaar, Joseph McKenna, and John Runions for seed stocks and vectors. This work was supported by BBSRC grants BB/M024172/1 and BB/H017569/1 and Royal Society equipment grant RG140321.

AUTHOR CONTRIBUTIONS

Conceptualization, M.J.D., P.J.H., and B.D.; Methodology, M.J.D. and S.S.; Investigation, M.J.D., S.S., C.R., S.W.M., A.N., E.A., and G.R.L.; Writing – Original Draft, M.J.D. and S.S.; Writing – Review & Editing, M.J.D., S.S., P.J.H., N.J.T., C.S., and B.D.; Funding Acquisition, M.J.D., C.S., and P.J.H.; Supervision, M.J.D., P.J.H., B.D., C.S., and N.J.T.

DECLARATION OF INTERESTS

The authors declare no competing interests.

Received: September 10, 2017
Revised: March 21, 2018
Accepted: May 4, 2018
Published: June 21, 2018

REFERENCES

1. Kobayashi, Y., Kobayashi, I., Funaki, Y., Fujimoto, S., Takemoto, T., and Kunoh, H. (1997). Dynamic reorganization of microfilaments and microtubules is necessary for the expression of non-host resistance in barley coleoptile cells. Plant J. *11*, 525–537.
2. Kobayashi, Y., Yamada, M., Kobayashi, I., and Kunoh, H. (1997). Actin microfilaments are required for the expression of nonhost resistance in higher plants. Plant Cell Physiol. *38*, 725–733.
3. Li, J., Henty-Ridilla, J.L., Staiger, B.H., Day, B., and Staiger, C.J. (2015). Capping protein integrates multiple MAMP signalling pathways to modulate actin dynamics during plant innate immunity. Nat. Commun. *6*, 7206.
4. Takemoto, D., Jones, D.A., and Hardham, A.R. (2003). GFP-tagging of cell components reveals the dynamics of subcellular re-organization in response to infection of Arabidopsis by oomycete pathogens. Plant J. *33*, 775–792.
5. Overdijk, E.J.R., DE Keijzer, J., DE Groot, D., Schoina, C., Bouwmeester, K., Ketelaar, T., and Govers, F. (2016). Interaction between the moss *Physcomitrella patens* and *Phytophthora*: a novel pathosystem for live-cell imaging of subcellular defence. J. Microsc. *263*, 171–180.
6. Chowdhury, J., Henderson, M., Schweizer, P., Burton, R.A., Fincher, G.B., and Little, A. (2014). Differential accumulation of callose, arabinoxylan and cellulose in nonpenetrated versus penetrated papillae on leaves of barley infected with *Blumeria graminis f. sp. hordei*. New Phytol. *204*, 650–660.
7. Miklis, M., Consonni, C., Bhat, R.A., Lipka, V., Schulze-Lefert, P., and Panstruga, R. (2007). Barley MLO modulates actin-dependent and actin-independent antifungal defense pathways at the cell periphery. Plant Physiol. *144*, 1132–1143.
8. Deeks, M.J., Fendrych, M., Smertenko, A., Bell, K.S., Oparka, K., Cvrcková, F., Zársky, V., and Hussey, P.J. (2010). The plant formin AtFH4 interacts with both actin and microtubules, and contains a newly identified microtubule-binding domain. J. Cell Sci. *123*, 1209–1215.
9. Deeks, M.J., Cvrcková, F., Machesky, L.M., Mikitová, V., Ketelaar, T., Zársky, V., Davies, B., and Hussey, P.J. (2005). Arabidopsis group Ie formins localize to specific cell membrane domains, interact with actin-binding proteins and cause defects in cell expansion upon aberrant expression. New Phytol. *168*, 529–540.
10. Humphry, M., Bednarek, P., Kemmerling, B., Koh, S., Stein, M., Göbel, U., Stüber, K., Pislewska-Bednarek, M., Loraine, A., Schulze-Lefert, P., et al. (2010). A regulon conserved in monocot and dicot plants defines a functional module in antifungal plant immunity. Proc. Natl. Acad. Sci. USA *107*, 21896–21901.
11. Shekhar, S., Pernier, J., and Carlier, M.F. (2016). Regulators of actin filament barbed ends at a glance. J. Cell Sci. *129*, 1085–1091.
12. Michelot, A., Derivery, E., Paterski-Boujemaa, R., Guérin, C., Huang, S., Parcy, F., Staiger, C.J., and Blanchoin, L. (2006). A novel mechanism for the formation of actin-filament bundles by a nonprocessive formin. Curr. Biol. *16*, 1924–1930.
13. Gurel, P.S., Ge, P., Grintsevich, E.E., Shu, R., Blanchoin, L., Zhou, Z.H., Reisler, E., and Higgs, H.N. (2014). INF2-mediated severing through actin filament encirclement and disruption. Curr. Biol. *24*, 156–164.
14. Cvrcková, F. (2000). Are plant formins integral membrane proteins? Genome Biol. *1*, research001.1–research001.7.
15. Deeks, M.J., Hussey, P.J., and Davies, B. (2002). Formins: intermediates in signal-transduction cascades that affect cytoskeletal reorganization. Trends Plant Sci. *7*, 492–498.
16. Stein, M., Dittgen, J., Sánchez-Rodríguez, C., Hou, B.H., Molina, A., Schulze-Lefert, P., Lipka, V., and Somerville, S. (2006). Arabidopsis PEN3/PDR8, an ATP binding cassette transporter, contributes to nonhost resistance to inappropriate pathogens that enter by direct penetration. Plant Cell *18*, 731–746.
17. Collins, N.C., Thordal-Christensen, H., Lipka, V., Bau, S., Kombrink, E., Qiu, J.L., Hückelhoven, R., Stein, M., Freialdenhoven, A., Somerville, S.C., and Schulze-Lefert, P. (2003). SNARE-protein-mediated disease resistance at the plant cell wall. Nature *425*, 973–977.
18. Underwood, W., and Somerville, S.C. (2013). Perception of conserved pathogen elicitors at the plasma membrane leads to relocalization of the Arabidopsis PEN3 transporter. Proc. Natl. Acad. Sci. USA *110*, 12492–12497.
19. Park, J.Y., Jin, J., Lee, Y.W., Kang, S., and Lee, Y.H. (2009). Rice blast fungus (*Magnaporthe oryzae*) infects Arabidopsis via a mechanism distinct from that required for the infection of rice. Plant Physiol. *149*, 474–486.
20. Mentlak, T.A., Kombrink, A., Shinya, T., Ryder, L.S., Otomo, I., Saitoh, H., Terauchi, R., Nishizawa, Y., Shibuya, N., Thomma, B.P., and Talbot, N.J. (2012). Effector-mediated suppression of chitin-triggered immunity by *magnaporthe oryzae* is necessary for rice blast disease. Plant Cell *24*, 322–335.
21. Jarosch, B., Jansen, M., and Schaffrath, U. (2003). Acquired resistance functions in mlo barley, which is hypersusceptible to *Magnaporthe grisea*. Mol. Plant Microbe Interact. *16*, 107–114.
22. Underwood, W., and Somerville, S.C. (2008). Focal accumulation of defences at sites of fungal pathogen attack. J. Exp. Bot. *59*, 3501–3508.
23. Xin, X.-F., Nomura, K., Underwood, W., and He, S.Y. (2013). Induction and suppression of PEN3 focal accumulation during *Pseudomonas syringae pv. tomato* DC3000 infection of Arabidopsis. Mol. Plant Microbe Interact. *26*, 861–867.
24. Lu, X., Dittgen, J., Piślewska-Bednarek, M., Molina, A., Schneider, B., Svatoš, A., Doubský, J., Schneeberger, K., Weigel, D., Bednarek, P., et al. (2015). Mutant Allele-Specific Uncoupling of PENETRATION3 Functions Reveals Engagement of the ATP-Binding Cassette Transporter in Distinct Tryptophan Metabolic Pathways. Plant Physiol. *168*, 814–827.
25. Yun, B.W., Atkinson, H.A., Gaborit, C., Greenland, A., Read, N.D., Pallas, J.A., and Loake, G.J. (2003). Loss of actin cytoskeletal function and EDS1 activity, in combination, severely compromises non-host resistance in Arabidopsis against wheat powdery mildew. Plant J. *34*, 768–777.

26. Smertenko, A.P., Deeks, M.J., and Hussey, P.J. (2010). Strategies of actin reorganisation in plant cells. J. Cell Sci. *123*, 3019–3028.

27. Huesmann, C., Reiner, T., Hoefle, C., Preuss, J., Jurca, M.E., Domoki, M., Fehér, A., and Hückelhoven, R. (2012). Barley ROP binding kinase1 is involved in microtubule organization and in basal penetration resistance to the barley powdery mildew fungus. Plant Physiol. *159*, 311–320.

28. Takemoto, D., Jones, D.A., and Hardham, A.R. (2006). Re-organization of the cytoskeleton and endoplasmic reticulum in the Arabidopsis *pen1-1* mutant inoculated with the non-adapted powdery mildew pathogen, *Blumeria graminis f. sp. hordei*. Mol. Plant Pathol. *7*, 553–563.

29. Sun, T., Li, S., and Ren, H. (2017). OsFH15, a class I formin, interacts with microfilaments and microtubules to regulate grain size via affecting cell expansion in rice. Sci. Rep. *7*, 6538.

30. Rosero, A., Žársky, V., and Cvrčková, F. (2013). AtFH1 formin mutation affects actin filament and microtubule dynamics in *Arabidopsis thaliana*. J. Exp. Bot. *64*, 585–597.

31. Drakakaki, G., van de Ven, W., Pan, S., Miao, Y., Wang, J., Keinath, N.F., Weatherly, B., Jiang, L., Schumacher, K., Hicks, G., and Raikhel, N. (2012). Isolation and proteomic analysis of the SYP61 compartment reveal its role in exocytic trafficking in Arabidopsis. Cell Res. *22*, 413–424.

32. Meyer, D., Pajonk, S., Micali, C., O'Connell, R., and Schulze-Lefert, P. (2009). Extracellular transport and integration of plant secretory proteins into pathogen-induced cell wall compartments. Plant J. *57*, 986–999.

33. Nielsen, M.E., Feechan, A., Böhlenius, H., Ueda, T., and Thordal-Christensen, H. (2012). Arabidopsis ARF-GTP exchange factor, GNOM, mediates transport required for innate immunity and focal accumulation of syntaxin PEN1. Proc. Natl. Acad. Sci. USA *109*, 11443–11448.

34. Henty-Ridilla, J.L., Li, J., Day, B., and Staiger, C.J. (2014). ACTIN DEPOLYMERIZING FACTOR4 regulates actin dynamics during innate immune signaling in Arabidopsis. Plant Cell *26*, 340–352.

35. Martinière, A., Gayral, P., Hawes, C., and Runions, J. (2011). Building bridges: formin1 of Arabidopsis forms a connection between the cell wall and the actin cytoskeleton. Plant J. *66*, 354–365.

36. Martinière, A., Lavagi, I., Nageswaran, G., Rolfe, D.J., Maneta-Peyret, L., Luu, D.-T., Botchway, S.W., Webb, S.E.D., Mongrand, S., Maurel, C., et al. (2012). Cell wall constrains lateral diffusion of plant plasma-membrane proteins. Proc. Natl. Acad. Sci. USA *109*, 12805–12810.

37. Samson, F., Brunaud, V., Balzergue, S., Dubreucq, B., Lepiniec, L., Pelletier, G., Caboche, M., and Lecharny, A. (2002). FLAGdb/FST: a database of mapped flanking insertion sites (FSTs) of *Arabidopsis thaliana* T-DNA transformants. Nucleic Acids Res. *30*, 94–97.

38. Marc, J., Granger, C.L.C., Brincat, J., Fisher, D.D., Kao, Th., McCubbin, A.G.A., and Cyr, R.J.R.R.J. (1998). A GFP-MAP4 reporter gene for visualizing cortical microtubule rearrangements in living epidermal cells. Plant Cell *10*, 1927–1940.

39. Clough, S.J., and Bent, A.F. (1998). Floral dip: a simplified method for Agrobacterium-mediated transformation of *Arabidopsis thaliana*. Plant J. *16*, 735–743.

40. Gutierrez, R., Lindeboom, J.J., Paredez, A.R., Emons, A.M.C., and Ehrhardt, D.W. (2009). Arabidopsis cortical microtubules position cellulose synthase delivery to the plasma membrane and interact with cellulose synthase trafficking compartments. Nat. Cell Biol. *11*, 797–806.

41. Eisinger, W.R., Kirik, V., Lewis, C., Ehrhardt, D.W., and Briggs, W.R. (2012). Quantitative changes in microtubule distribution correlate with guard cell function in Arabidopsis. Mol. Plant *5*, 716–725.

42. van der Honing, H.S., Kieft, H., Emons, A.M., and Ketelaar, T. (2012). Arabidopsis VILLIN2 and VILLIN3 are required for the generation of thick actin filament bundles and for directional organ growth. Plant Physiol. *158*, 1426–1438.

43. Sessions, A., Burke, E., Presting, G., Aux, G., McElver, J., Patton, D., Dietrich, B., Ho, P., Bacwaden, J., Ko, C., et al. (2002). A high-throughput Arabidopsis reverse genetics system. Plant Cell *14*, 2985–2994.

44. Littlejohn, G.R., Mansfield, J.C., Christmas, J.T., Witterick, E., Fricker, M.D., Grant, M.R., Smirnoff, N., Everson, R.M., Moger, J., and Love, J. (2014). An update: improvements in imaging perfluorocarbon-mounted plant leaves with implications for studies of plant pathology, physiology, development and cell biology. Front. Plant Sci. *5*, 140.

45. Talbot, N.J., Ebbole, D.J., and Hamer, J.E. (1993). Identification and characterization of MPG1, a gene involved in pathogenicity from the rice blast fungus *Magnaporthe grisea*. Plant Cell *5*, 1575–1590.

46. Mascarenhas, J.P., and Hamilton, D.A. (1992). Artifacts in the localization of GUS activity in anothers of petunia transformed with a CaMV 35S-GUS construct. Plant J. *2*, 405–408.

47. Faris, J.D., Zhang, Z., Lu, H., Lu, S., Reddy, L., Cloutier, S., Fellers, J.P., Meinhardt, S.W., Rasmussen, J.B., Xu, S.S., et al. (2010). A unique wheat disease resistance-like gene governs effector-triggered susceptibility to necrotrophic pathogens. Proc. Natl. Acad. Sci. USA *107*, 13544–13549.

48. Rasmussen, R. (2001). Quantification on the LightCycler. In Rapid Cycle Real-Time PCR: Methods and Applications, S. Meuer, C. Wittmer, and K. Nakagawara, eds. (Springer Berlin Heidelberg), pp. 21–34.

49. Filby, A.L., and Tyler, C.R. (2005). Molecular characterization of estrogen receptors 1, 2a, and 2b and their tissue and ontogenic expression profiles in fathead minnow (*Pimephales promelas*). Biol. Reprod. *73*, 648–662.

50. Schindelin, J., Arganda-Carreras, I., Frise, E., Kaynig, V., Longair, M., Pietzsch, T., Preibisch, S., Rueden, C., Saalfeld, S., Schmid, B., et al. (2012). Fiji: an open-source platform for biological-image analysis. Nat. Methods *9*, 676–682.

51. Wang, R., Okamoto, M., Xing, X., and Crawford, N.M. (2003). Microarray analysis of the nitrate response in Arabidopsis roots and shoots reveals over 1,000 rapidly responding genes and new linkages to glucose, trehalose-6-phosphate, iron, and sulfate metabolism. Plant Physiol. *132*, 556–567.

52. Hruz, T., Laule, O., Szabo, G., Wessendorp, F., Bleuler, S., Oertle, L., Widmayer, P., Gruissem, W., and Zimmermann, P. (2008). Genevestigator v3: a reference expression database for the meta-analysis of transcriptomes. Adv. Bioinforma. *2008*, 420747.

53. Nelson, C.J., Duckney, P., Hawkins, T.J., Deeks, M.J., Laissue, P.P., Hussey, P.J., and Obara, B. (2014). Blobs and curves: object-based colocalisation for plant cells. Funct. Plant Biol. *42*, 471–485.

54. Obara, B., Jabeen, A., Fernandez, N., and Laissue, P.P. (2013). A novel method for quantified, superresolved, three-dimensional colocalisation of isotropic, fluorescent particles. Histochem. Cell Biol. *139*, 391–402.

STAR★METHODS

KEY RESOURCES TABLE

REAGENT or RESOURCE	SOURCE	IDENTIFIER
Bacterial and Virus Strains		
Escherichia coli DH5α	N/A	N/A
Agrobacterium tumefaciens GV3101	N/A	N/A
Chemicals, Peptides, and Recombinant Proteins		
Murashige and Skoog (MS) basal medium with Gamborg's Vitamins	Sigma-Aldrich	Cat#M0404
Nutrient Agar	LabM	Cat#LAB008
Levington F2 + Sand multipurpose compost	JFC Munro	Cat# LEV206
Vermiculite Medium	JFC Munro	Cat#VER016
Flutec PP-11 (Perfluoroperhydrophenanthrene)	F2 Chemicals Ltd	Cat#306-91-2
Aniline blue	Sigma-Aldrich	Cat#415049
X-GlcA (5-Bromo-4-chloro-3-indolyl-β-D-glucuronic acid, cyclohexyl ammonium salt)	Melford Biolaboratories Ltd	Cat#MB1021
Triton X-100	Sigma-Aldrich	Cat#T8787
Potassium hexacyanoferrate(II) trihydrate	Sigma-Aldrich	Cat#P3289
Potassium hexacyanoferrate(III)	Sigma-Aldrich	Cat#244023
Glycerol	Sigma-Aldrich	Cat#G5516
RQ1 RNase-Free DNase	Promega	Cat#M6101
M-MLV reverse transcriptase	Promega	Cat#M5313
iTaq Universal SYBR Green Supermix	Bio-Rad Laboratories	Cat#1725120
Chitin from crab shells	Sigma-Aldrich	Cat#417955
Driselase from *Basidiomycete sp.*	Sigma-Aldrich	Cat#D8037
Chitinase from *Trichoderma viride*	Sigma-Aldrich	Cat#C8241
Borosilicate class coverslips D263M	ThermoScientific	Cat#BB02200500A033MNT0
MES Monohydrate [2-(N-Morpholino)-ethanesulfonic acid]	Melford	Cat#M22040
Caffeic acid	Sigma-Aldrich	Cat#C0625
Microscope slides	Thermo Scientific	Cat#AD00000102E00MNT10
Surgical tape, Micropore 3M	VWR	Cat#115-8172
Latrunculin A	Sigma-Aldrich	Cat#428020
Latrunculin B	Sigma-Aldrich	Cat#L5288
DMSO	Sigma-Aldrich	Cat#M81802
Oryzalin	Sigma-Aldrich	Cat#36182
Cytochalasin E	Sigma-Aldrich	Cat#C8273
D-Sorbitol	Sigma-Aldrich	Cat#S1876
FM4-64 Dye (N-(3-Triethylammoniumpropyl)-4-(6-(4-(Diethylamino) Phenyl) Hexatrienyl) Pyridinium Dibromide)	ThermoFisher Scientific	Cat#T13320
Critical Commercial Assays		
RNeasy Plant Mini Kit	QIAGEN	Cat#74904
Qubit RNA IQ Assay Kit	ThermoFisher Scientific	Cat#Q33222
GeneJET Plasmid Miniprep Kit	ThermoFisher Scientific	Cat#K0503
Deposited Data		
Genevestigator gene expression data	Genevestigator	ATH1 genome array data
Experimental Models: Organisms/Strains		
Blumeria graminis f. sp. *Hordei*: UK isolate CC/133	NIAB	N/A
Hordeum vulgare: Variety "Golden Promise"	N/A	N/A

(*Continued on next page*)

Continued

REAGENT or RESOURCE	SOURCE	IDENTIFIER
Magnaporthe oryzae: Strain Guy-11	N/A	N/A
Arabidopsis: Col-0	Lehle Seeds	Cat#WT-02
Arabidopsis: Ws	Lehle Seeds	Cat#WT-8A
Arabidopsis: *formin4-1* (At1g24150)	[37]	N/A
Arabidopsis: *formin7-1* (At1g59910)	[43] NASC	NASC ID: N879635
Arabidopsis: *formin8-1* (At1g70140)	This study	N/A
Arabidopsis: *formin4/7/8*	This study	N/A
Arabidopsis: GFP-MAP4	[38]	N/A
Arabidopsis: *pen3-1*: PEN3-GFP	[16]	NASC ID: N67802
Arabidopsis: GFP-Lifeact (Col-0)	[26]	**N/A**
Arabidopsis: GFP-Lifeact (*formin4/7/8*)	This study	N/A
Arabidopsis: mCherry-TUA5 (Col-0)	Tijs Ketelaar, University of Wageningen	N/A
Arabidopsis: mCherry-TUA5 (*formin4/7/8*)	This study	N/A
Arabidopsis: formin4/7/8: FORMIN4-GFP	This study	N/A
Arabidopsis: FORMIN4-GFP	This study	N/A
Arabidopsis: formin4/7/8: FORMIN4(ΔFH1-FH2)-GFP	This study	N/A
Arabidopsis: FORMIN4(ΔFH1-FH2)-GFP	This study	N/A
Arabidopsis: FORMIN4tdTomato/PEN3-GFP (*pen3-1*)	This study	N/A
Oligonucleotides		
FH4GUSF: GGGGACAAGTTTGTACAAAAAAGCAGGCTTA TCAATACAAGAAGTCAAGAAGAAGACGTG	This study	N/A
FH4GUSR: GGGGACCACTTTGTACAAGAAAGCTGGGT TGGAAGATTAACTCATTTGTTTAGAG	This study	N/A
FH4RA: GGGGACCACTTTGTACAAGAAAGCTGGGTTCA CATATCGGAATCTGATCCACCCG	This study	N/A
QFH4FA: TTCAGGGGAAAGTTCAAATGGTCAG	This study	N/A
QFH4RA: TTTTATCACCGCCGTCGTCT	This study	N/A
QFH4FB: ACTCAGTTCCGTTATACACAG	This study	N/A
QFH4RB: TTTTATCACCGCCGTCGTCT	This study	N/A
PTB1F: TTCAGGGGAAAGTTCAAATGGTCAG	This study	N/A
PTB1R: TTTTATCACCGCCGTCGTCT	This study	N/A
FH4GENFA: TTGATGCAGCCATGGCCACCG	This study	N/A
FH4GENRA: AAAGAATCAGTCTTGACATGAGGAG	This study	N/A
FH4GENRB: GATATAACCTCGGAGATCGAACTGC	This study	N/A
FH7GENFA: AAGAACGGTAGTAGTTCACGGAGGAAG	This study	N/A
FH7GENRA: CCACCATAATCACTACCGGCACTTGT	This study	N/A
FH8GENFA: CTACAGAGTCAGAGAGAAAGAAGTG	This study	N/A
FH8GENRA: TTTCTCTTGCTCTTCTTTCGACATAAC	This study	N/A
TAG3: CTGATACCAGACGTTGCCCGCATAA	This study	N/A
LB3: TAGCATCTGAATTTCATAACCAATCTCGATACAC	This study	N/A
QFH7FA:ACTTCTCACAGTGTTATCCATAACGAAG	This study	N/A
QFH7RA:TGAAACGAAAACGCCTCTTCGATAG	This study	N/A
QFH8FA:ACTTCTCACAGTGTTATCCATAACGAAG	This study	N/A
QFH8RA:CTCTCCTCCACTTGCTCCTCT	This study	N/A
EF1F:CCCATTTGTGCCCATCTCT	This study	N/A
EF1R:CACCGTTCCAATACCACCAA	This study	N/A
Recombinant DNA		
Plasmid: pFormin4::FORMIN4	This study	N/A
Plasmid: pFormin4::FORMIN4(ΔFH1-FH2)	This study	N/A

(*Continued on next page*)

Continued		
REAGENT or RESOURCE	SOURCE	IDENTIFIER
Plasmid: pFormin4::uidA	This study	N/A
Plasmid: pDONR207	Invitrogen	N/A
Plasmid: pBI101G	Martin Kieffer and Brendan Davies, University of Leeds	N/A
Plasmid: FORMIN4-tdTomato	This study	N/A
Plasmid: pB7FWGtdTomato	Joeseph McKenna and John Runions, Oxford Brookes University	N/A
Plasmid: pH7FWG	VIB, Gent	N/A
Plasmid: GFP-Lifeact	[26]	N/A
Plasmid: mCherry-TUA5	Tijs Ketelaar, University of Wageningen	N/A
Software and Algorithms		
Beacon Designer 7	Premier Biosoft International, Palo Alto, USA	http://www.premierbiosoft.com/molecular_beacons/
ImageJ	National Institute of Health, USA	http://imagej.net
Fiji	National Institute of Health, USA	https://fiji.sc/
LAS-X software	Leica	https://www.leica-microsystems.com/
Photoshop CS6	Adobe Systems	https://www.adobe.com
Illustrator CS4	Adobe Systems	https://www.adobe.com
SPSS 23.0	SPSS	https://www.ibm.com/
Inkscape 0.92	Inkscape Project	https://inkscape.org/en/
VisiView	Visitron Systems GmbH	http://www.visitron.de/Products/Software/VisiView/visiview.html
MATLAB 2016b	The MathWorks, Natick, MA, USA	https://www.mathworks.com
Excel	Microsoft	https://products.office.com/en-gb/excel
Word	Microsoft	https://products.office.com/en-gb/word
Genevestigator application	Nebion	https://genevestigator.com/gv/

CONTACT FOR REAGENT AND RESOURCE SHARING

Further information and requests for resources and reagents should be directed to and will be fulfilled by the Lead Contact, Mike Deeks (m.deeks@exeter.ac.uk).

EXPERIMENTAL MODEL AND SUBJECT DETAILS

Plant material

Arabidopsis thaliana (L. Hyhn) ecotype Columbia-0 (Col-0) was used as the background for recombinant lines. Stable *A. thaliana* transformants were produced by floral dipping using Agrobacterium (GV3 101) with the method described by Clough and Bent [39]. PEN3-GFP in a rescued *pen3-1* mutant [16] was a gift of Shauna Somerville (UC Berkeley, USA). The GFP-Lifeact and Col-0 transformant line were described previously by Smertenko et al. [26]. The mCherry-TUA5 Col-0 line and plasmid were a gift from Tijs Ketelaar and contains an alpha-tubulin 5 Gateway-compatible coding sequence [40] in an mCherry derivative of pMDC43 [41, 42]. The GFP-MAP4 line and construct has previously been described by Marc et al. [38] All transgenic lines were derived from true-breeding T2 plants. Exceptions were lines ‘Comp. 2′ and ‘Comp. 3′ (Figure S3) where segregating T2 individuals were screened for FORMIN4-GFP fluorescence. The *formin4/7/8* mutant was assembled from T-DNA insert alleles *formin4-1* (At1g24150, FLAG allele [37] gifted by Frederic Berger, Gregor Mendel Institute, Austria), *formin7-1* (At1g59910, SAIL line 677E8 [43]) and *formin8-1* (At1g70140, SAIL line 93D11). Homozygous T-DNA insertion lines were selected using PCR primers for details see resources table and Table S1.

Plant growth conditions

A. thaliana seeds were surface fume sterilized in a sealed container with 100 mL bleach supplemented by 3 mL of 37% HCl (to produce chlorine gas) for 4-5 h, then suspended in molecular biology grade water and stratified at 4°C for at least 4 days. For genetic analysis and imaging of leaves plants were grown in 16 hours of light (150 μmol/m^2/s) at 21°C, with an 8-hour dark period at 19°C. After stratification *A. thaliana* seeds for dark grown elongated hypocotyls, were placed on 100 μl half-concentration Murashige and Skoog (MS) basal medium with Gamborg's Vitamins (Sigma-Aldrich, UK) containing 0.8% w/v agar (Lab M, UK). 500 μl PCR tubes containing the media were used as a supportive environment for the growing hypocotyls. These were placed within a humid chamber and plants were grown in darkness at 21°C over a period of five days. This workflow was designed to ensure sterility and to minimize mechanical disruption.

Pathogen growth conditions

Blumeria graminis f. sp. *hordei* (*Bgh*), UK isolate CC/133 (NIAB, Cambridge, UK) was cultivated on *Hordeum vulgare* L. variety "Golden Promise" (8 h photoperiod, 120 μmol/m^2, at 17°C) by weekly infection of three-week old barley plants.

Powdery mildew infection assay – growth conditions

Seeds were stratified in darkness, on soil (Levington F2 + Sand multipurpose compost mixed 3:1 v/v with Vermiculite), at 4°C for 7 days. Plants were grown for 2 to 2 ½ weeks. After the cultivation period *Bgh*-spores were sprinkled on whole seedlings. Inoculated plants were completely shaded and kept at 17°C for 48 h.

METHOD DETAILS

Powdery mildew infection assay

First true leaves with an evaluated spore density of 60 (±35) spores per mm^2 were cut and mounted on slides in Flutec PP-11 (Perfluoroperhydrophenanthrene, F2 Chemicals, UK) immediately before observation. PP-11, a low surface tension solvent compatible with live-cell imaging [44], enabled observation without any staining, clearing or vacuum infiltration. We compared aniline blue labeling of whole-cell callose encasement to transmission microscopy (with PP11 mounting solution) as methods for identifying leaf epidermal cells undergoing lethal immune responses in the *formin4/7/8* triple mutant. In the case of transmission microscopy these were cells with aggregated cytoplasm, arrested cytoplasmic streaming and thickened cell walls. Of the investigated epidermal cells (n = 54), 22 out of 23 encased cells identified using aniline blue were identified by transmission microscopy (one encased cell remained active with indications of cytoplasmic streaming). No false-positive encased cells were detected using transmission microscopy. We therefore chose to use transmission microscopy to identify dead cells as this required less preparation and risk of misinterpretation of excessive callose production for cell death. We defined four categories of response to *Bgh* based on our initial observations. These were 'CWA response + no haustorium + cytoplasmic streaming', 'CWA response + no haustorium + cell death', 'haustorium + cytoplasmic streaming' and 'haustorium + cell death'. 20.2% of host cells with haustoria showed cytoplasmic streaming and this proportion was not found to vary significantly (using Fisher's exact test) between genotypes. Consequently we have summed these two 'haustoria' categories in Figure 3 and Figure S3. The actin filament phenotype (Figure 3G,H) was scored using three biological repeats, with a minimum of three leaves per genotype per repeat and 10 CWAs per leaf.

Magnaporthe oryzae infection

Strain Guy-11 of *M. oryzae* was maintained and prepared for infection as described [45]. In order to infect *A. thaliana* dark-grown hypocotyl cells it was necessary to apply conidia from suspension using a clean cotton bud. Samples were imaged 16 hours post-infection.

Plasmids

The pFormin4::uidA transcriptional fusion of the uidA gene for the *FORMIN4* promoter was produced by amplifying a 1,512 bp fragment containing the promoter and 5-prime UTR of *FORMIN4* (At1g24150) from *A. thaliana* Col-0 genomic DNA with primers FH4GUSF and FH4GUSR. These primers include attB sequences compatible with the Gateway recombination system (Invitrogen). The fragment was recombined into entry vector pDONR207 (Invitrogen), sequenced and recombined into destination vector pBI101G containing the uidA gene (a Gateway conversion of pBI101; provided by Martin Kieffer, University of Leeds UK). The pFormin4::FORMIN4 and pFormin4::FORMIN4(ΔFH1-FH2) were amplified from genomic DNA using reverse primers FH4RA and FH4RB respectively, combined with forward primer FH4GUSF. Both were cloned into entry vector pDON207. Translational fusions to GFP were achieved by recombining into pH7FWG (VIB, Gent, Belgium) and FORMIN4-tdTomato was produced by recombining into a tdTomato conversion of pB7FWG (provided by Joe McKenna and John Runions, Oxford Brookes University, UK).

GUS Analysis

Beta-glucuronidase (GUS) activity was detected using the method described in [46]. Briefly, leaves were incubated in 0.1 M sodium phosphate buffer, pH 7 with 500 μg/ml X-GlcA (5-Bromo-4-chloro-3-indolyl-β-D-glucuronic acid, cyclohexyl ammonium salt, Melford, UK) and 0.1% Triton X-100. 1 mM potassium ferrocyanide and 1 mM potassium ferricyanide were included in the buffer

as catalysts. Submerged tissue was vacuum infiltrated and incubated overnight at 37°C. Chlorophyll was removed from the samples using an ethanol series before the tissue was rehydrated. Whole-mounting was performed in 40% v/v glycerol.

RNA purification and reverse transcriptase

Whole RNA was isolated from single infected seedlings two days after infection using the RNeasy Plant Mini Kit (QIAGEN, Manchester, UK) according to the manufacturer's guidelines. After determining the total amount of RNA using the Qubit 4 Fluorometer (Thermo Fisher Scientific, Waltham, MA, USA) 300 ng total RNA of each sample were treated with RQ1 RNase-Free DNase (Promega, Southampton, UK) and cDNA was synthesized using random hexamers and M-MLV reverse transcriptase (Promega, UK).

RT-qPCR

Relative transcript concentration of *FORMIN4* was determined by quantitative real-time PCR (RT-qPCR). Primers for the house-keeping gene (*ptb1*; AT3G01150) were designed using the Beacon Designer 7 software (Premier Biosoft International, Palo Alto, USA), primers for elongation factor one alpha (AT5G60390) and primers for *FORMIN4* were designed manually, using full length cDNA and genomic sequences (http://www.arabidopsis.org). For the quantitative experiments shown in Figure S3, panels B and C, the annealing temperature for each primer pair was optimized by running a temperature gradient program followed by a melt curve analysis to verify primer specificity. To determine the detection range, as well as linearity and RT-qPCR amplification efficiency of the primer pairs, assays were run in triplicates on serial dilutions; for the house-keeping gene 10-fold and for *FORMIN4* twofold on sample cDNA. A standard curve (mean threshold cycle (Ct) versus log cDNA dilution) gives the slope, which can be translated into high efficiency E ($E = 10^{(-1/\mathrm{slope})}$) [47] [48]. The linear correlation (R^2) of the mean Ct and the log cDNA dilution over the detection range was > 0.99, with a slope of 3.892 for QFH4FB/RB and 3.428 for the house-keeping gene.

For the RT-qPCR cDNA samples were diluted 1:3 and the reaction was performed with a CFX Connect Real-time PCR detection system (Bio-Rad Laboratories, Inc., Hercules, CA, USA). Each sample was amplified in triplicates in a 15 μl reaction using 1 μl cDNA in 1x iTaq Universal SYBR Green Supermix (Bio-Rad Laboratories, Inc., Hercules, CA, USA) running 30 cycles, followed by a melt curve analysis to validate one specific PCR product.

The relative gene expression (*RE*) was calculated according to [49] as $RE = (E_{\mathrm{ref}})^{\mathrm{Ct}} / (E_{\mathrm{target}})^{\mathrm{Ct}}$.

Hypocotyl elicitation and imaging

To trigger an immune response in *A. thaliana* elongated hypocotyls we exposed seedlings to an elicitor solution. This elicitor was chitin granules (100 μg/ml) from crab shells (Sigma Aldrich, UK), 0.004% w/v driselase (crude extract from basidiomycetes sp; Sigma Aldrich, UK) and 0.3 units endochitinase /ml from a stock solution containing endochitinase from *Trichoderma viride* (Sigma Aldrich, UK) dissolved in 100 mM sodium phosphate buffer at pH 6.1 and 50% v/v glycerol. A mock stock buffer that excluded the endochitinase was produced for control treatments. After addition of the endochitinase solution the mix was incubated for 20 minutes at room temperature with regular inversion. Elicitor was applied four hours before observation by filling growth tubes with the solution.

Whole hypocotyls were mounted onto 32x50 mm D263M borosilicate class coverslips with 0.08-0.12 mm thickness (Menzel-Gläser, ThermoScientific, UK) and embedded in mounting media (MM) consisting of 10 mM MES buffer at pH 7.5 (KOH/HCl), 1 mg/L caffeic acid and molecular pattern elicitor mix (1:10 v/v). When applicable, drug concentration was kept constant in the MM. Coverslips were mounted on microscope slides 76 × 26 mm with 90° ground edges (Menzel-Gläser, Thermo Scientific, UK), and gently fastened with micro pore tape (3M, UK) to ensure close contact of the hypocotyl with the coverslip.

Imaging was performed on a variable angle inverted TIRF microscope Olympus IX81 using a PlanApo 100x TIRF Oil objective lens with a numerical aperture of 1.45. Angles of incidence of the solid state laser (Coherent, USA) at peak emission of 488 nm (10% of CW: 50 mW) were optimized for each sample to maximize contrast. The differentiation of GFP signal from possible auto fluorescence was achieved by a dual beam splitter Photometrics®DV2 (Photometrics, USA) which enabled us to simultaneously image the fluorescence from 505-545 nm (eGFP filter set, Chroma Technology Corporation, USA) and 610-645 nm (TxRED HC-filter set, Chroma Technology Corporation, USA) and record both fluorescent channels by a CoolSNAP HQ2 CCD camera. (Visitron systems, Germany).

For fluorescence recovery investigations an area of 64x64 pixel (17.04 μm) was bleached using a 2D-VisiFRAP Realtime Scanner (Visitron Systems, Germany) by a diode pumped green crystal laser (CrystaLaser, USA) at 405 nm (70% of CW: 120 mW) over a total period of 8.45 s (50 ms/pixel). To minimize any damaging effects of the bleach laser its intensity was kept to a minimum which still achieved a total bleach of the region of interest (ROI).

Sites were imaged for 4 s periods consisting of 40 images (100 ms exposure time each). Time series up to 10 minutes were recorded with an interval every minute including one before and one immediately after the photo bleach. Total observation time for each sample under the microscope was limited to a maximum of 30 minutes. Mock induction of the immune response without elicitor mix showed no accumulation of FORMIN4-GFP after 4 h.

Drug treatments

Actin polymerization within *A. thaliana* hypocotyl cells was inhibited by application of 10 μM Latrunculin (A or B as specified; Sigma-Aldrich, UK) from 10 mM stock (in DMSO) for 30 min in the molecular pattern mix. To control for effects of DMSO a mock drug treatment with the respective amount of DMSO in the elicitation solution and MM was performed over the same treatment and observation period. Oryzalin stocks were made in DMSO at a concentration of 100 mM and used at a working concentration of 100 μM.

Cytochalasin E drug treatment of leaves was based on the protocol of Kobayashi et al. [1]. Cytochalasin E stocks were dissolved in DMSO at 5 mg ml^{-1} and diluted to a working concentration of 5 μg ml^{-1}. The solution was vacuum infiltrated into leaves of plants 2.5 weeks after germination. Infiltrations were also performed of a mock solution with an equivalent dilution of DMSO. After infiltration the leaves were immediately infected with *Bgh*.

Leaf plasmolysis

Leaves from FORMIN4-GFP transformants were infected with *Bgh* for 48 hours and then vacuum infiltrated with a solution of 5 M D-sorbitol [32]. The samples were mounted immediately in 5 M D-sorbitol and observed using confocal microscopy.

Aniline blue staining of callose deposits

Staining of hypocotyls was performed immediately before imaging to ensure elongated hypocotyl epidermal cells remained vital during imaging. Hypocotyls were mounted in aniline blue stain (0.1% w/v aniline blue in 150 mM K_2HPO_4 pH to 9.5). For leaf staining two days after *Bgh* infection, leaves were syringe infiltrated with aniline blue five minutes prior to imaging. Samples were only viewed for a maximum of 30 minutes to prevent additional cell death and aniline blue penetration into the cytoplasm. Images were acquired using sequential line scan between GFP settings (488 nm excitation and 505 to 530 nm emission) and aniline blue settings with 405 nm excitation combined with an emission window of 430 to 500 nm. Transmission, GFP and red channels (648 to 694 nm) were referenced to exclude any debris from the hypocotyl analysis (5 hypocotyls totalling 161 FORMIN4-GFP regions).

Infected leaf FM4-64 uptake

Plants lines were grown, stratified, and infected in the same conditions as for the powdery mildew assay. Prior to the experiment leaves were cut and attached to filter paper using micropore tape. After infection with *Bgh*-spores, leaves were kept shaded in a humid atmosphere at 17°C for 48 h.

Leaves were immersed in 1 μM FM4-64 (Molecular Probes, Eugene, USA) dye, freshly prepared in molecular biology grade water from a 1 mM Stock (in DMSO) for 90 minutes. Leaves were washed three times to remove any residual dye and mounted in water. Samples were imaged using an inverted Leica TCS SP8 confocal laser scanning microscope using a HCOL APO CS2 63x, NA 1.4 oil immersion lens. Samples were exited with an Argon AR+ML laser at 488 nm for simultaneous detection of GFP (detection window of 500-525 nm) and FM4-64 (630-700 nm) containing cellular compartments.

FRAP analysis

Experiments were performed in three biological repeats with a total sample size of elicitated and bleached regions of $n > 10$ for each treatment. Fluorescence images from TIRF and confocal microscopes were analyzed using the open source software FIJI, an image processing package distribution of ImageJ, (FIJI version 2.0.0-rc-44/1.5e / Java 1.8.0_66 [64bit] [50]. Further image and data processing was performed using the commercial software package MATLAB (R2016a_64bit, The Mathworks Inc., Natick, MA, 2016)

To analyze FRAP data, a square of 12.1 μm^2 was defined just within the border of each bleach zone to measure fluorescence recovery. Photo bleaching caused by the imaging laser was measured using neighboring zones that had not been affected by the initial bleach. After this correction was performed the signal intensity was normalized according to the values of the pre-bleach (100%) and first post-bleach (0%) time point to calculate a ‘percentage of recovery’ value. Recovery intensity in the first 5 minutes after bleach showed a linear behavior and was measured by linear regression using MATLAB.

We used a second method to measure recovery which we named SPRAP (spot recovery after photobleaching). Individual puncta were counted by recognizing local peaks of fluorescence using ‘regionprops’ in MATLAB. Images were pre-filtered for objects of appropriate size using a Laplacian of Gaussian (LoG) filter. SPRAP values were normalized to the spot numbers present in recovery zones immediately pre and post bleach.

Analysis of publicly available microarray data

Publicly available data generated using the Affymetrix ATH1 *A. thaliana* microarray (Affymetrix, Inc., Santa Clara, USA) [51] was analyzed using the Genevestigator application (Nebion, Switzerland) [52]. ‘Perturbation’ analyses were performed comparing 93 genes encoding members of well-characterized families of actin binding proteins. The impact of two classes of perturbations were measured; ‘biotic stimuli’ and ‘elicitors’. The output was filtered to remove comparisons that were not 1/ infections versus mock infections, 2/ elicitor versus mock elicitor, or 3/ time course measurements compared to either equivalent time points of mock treatments or to time zero. This resulted in 127 and 66 comparisons respectively for the two classes of perturbations. The 93 genes were scored on the basis of their frequency of upregulation or downregulation (defined as changes greater than a log2 value of 1; see Data S1).

Co-localization analysis

We analyzed three biological repeats of the FM4-64 uptake experiment with a minimum of 7 fungal interaction sites per repeat (each containing more than 15 images per site). An exception was the negative control showing only PEN3-GFP expression (this was performed three times). GFP and FM4-64 objects and their centroid positions were identified using the same method as SPRAP. Objects in each channel were paired using a Hungarian matching-algorithm [53, 54]. We defined that positive co-localization had occurred when the paired centroid distance was less than 200 nm, a value below the predicted Abbe limit. The amount of co-localization is

reported as the percentage of the total recognized FM4-64-labeled objects. Trend lines were added to histograms using a Gaussian kernel using the MATLAB ‘hisfit’ procedure with a ‘kernel’ setting and sigma value of 10.

QUANTIFICATION AND STATISTICAL ANALYSIS

Quantification of images was performed using FIJI, ImageJ and MATLAB. All statistical analysis and data plotting was performed using SPSS and MATLAB. For reasons of clarity the definition, number of N and used statistical test are stated at the appropriate methods section or figure caption. If not stated otherwise, data are presented as mean ± standard deviation (SD). The significant difference is defined as: * < 0.05; ** < 0.01; *** < 0.001 and is indicated by asterisks or different letters above the histogram bars.

Current Biology, Volume *28*

Supplemental Information

An Immune-Responsive Cytoskeletal-Plasma Membrane Feedback Loop in Plants

Stefan Sassmann, Cecilia Rodrigues, Stephen W. Milne, Anja Nenninger, Ellen Allwood, George R. Littlejohn, Nicholas J. Talbot, Christian Soeller, Brendan Davies, Patrick J. Hussey, and Michael J. Deeks

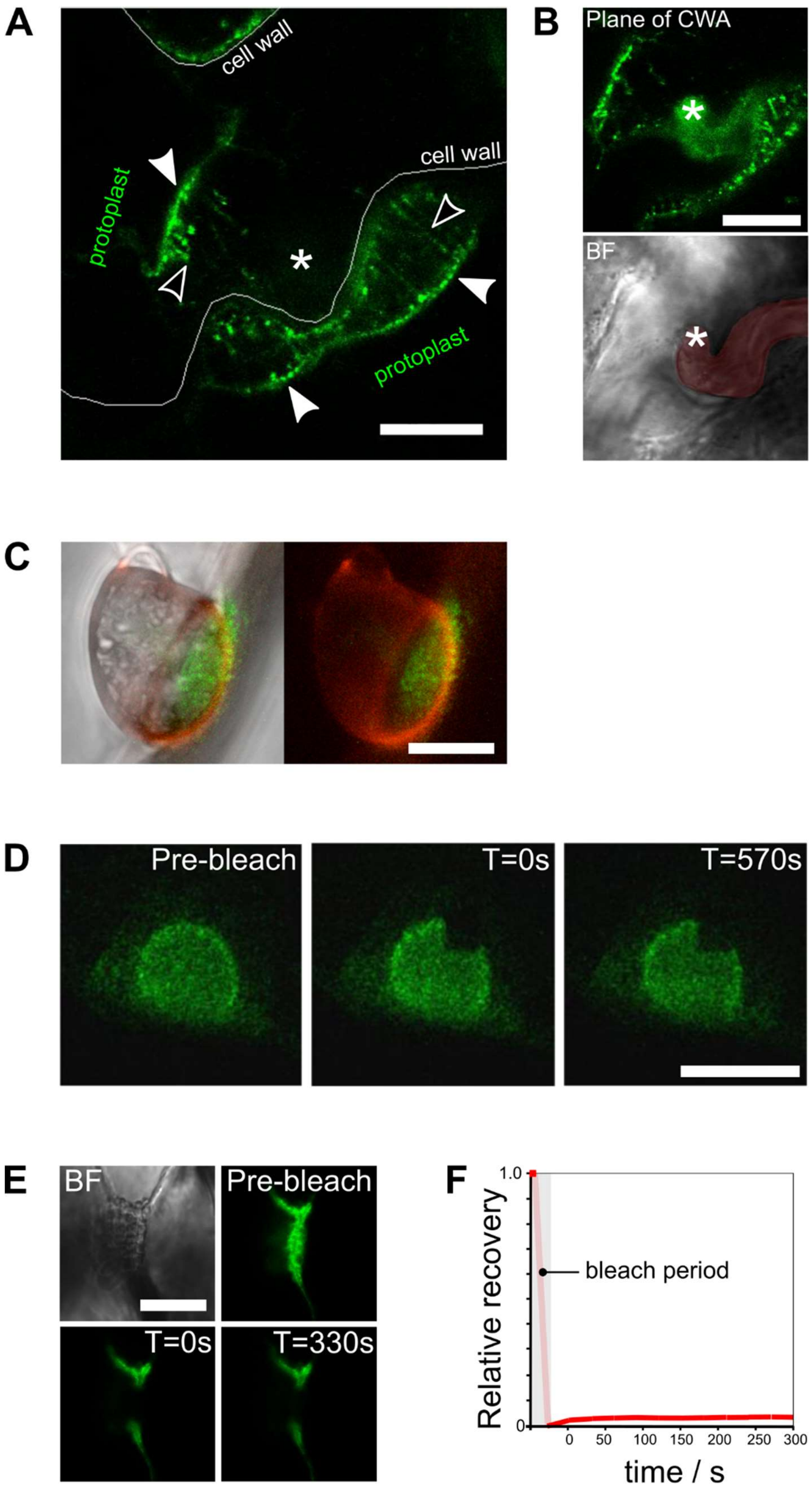

Figure S1. FORMIN4 accumulates in stable deposits at the plant plasma membrane in response to fungi, Related to Figure 1 and Video S1.

(A) Single optical sections of the plasmolysis of *Bgh*-infected epidermal cells expressing FORMIN4-GFP demonstrates that FORMIN4-GFP is associated with the plasma membrane rather than the apoplast. GFP fluorescence can be seen associated with the boundary of the shrinking protoplast. Enriched zones of FORMIN4-GFP membrane are marked with white arrowheads. Hechtian strands of membrane stretching from the cell wall to the protoplast labelled with FORMIN4-GFP are marked with black-filled arrow heads. The asterisk denotes the position of the CWA in the higher imaging plane. Scale bar is 10 µm.

(B) Image plane of the same cell containing the CWA. Asterisk indicates the position of the CWA. Scale bar is 10 µm.

(C) *A. thaliana* epidermal cell expressing FORMIN4-GFP (green channel) in response to a *Magnaporthe oryza* appressorium. Autofluorescence generated by the fungal cell wall can be seen in the red channel. Scale bar is 5 µm.

(D) FORMIN4-GFP discs induced by *Bgh* do not recover from the edges of the bleach zone after photobleaching. Scale bar is 10 µm.

(E) FORMIN4-GFP surrounding *Bgh*-induced CWAs in perpendicular cell walls were used to monitor the mobile GFP fraction during FRAP experiments. Scale bar is 10 µm.

(F) Typical example recovery curve taken from the CWA shown in (B), recording a negligible mobile fraction amounting to less than 5% of the original fluorescence.

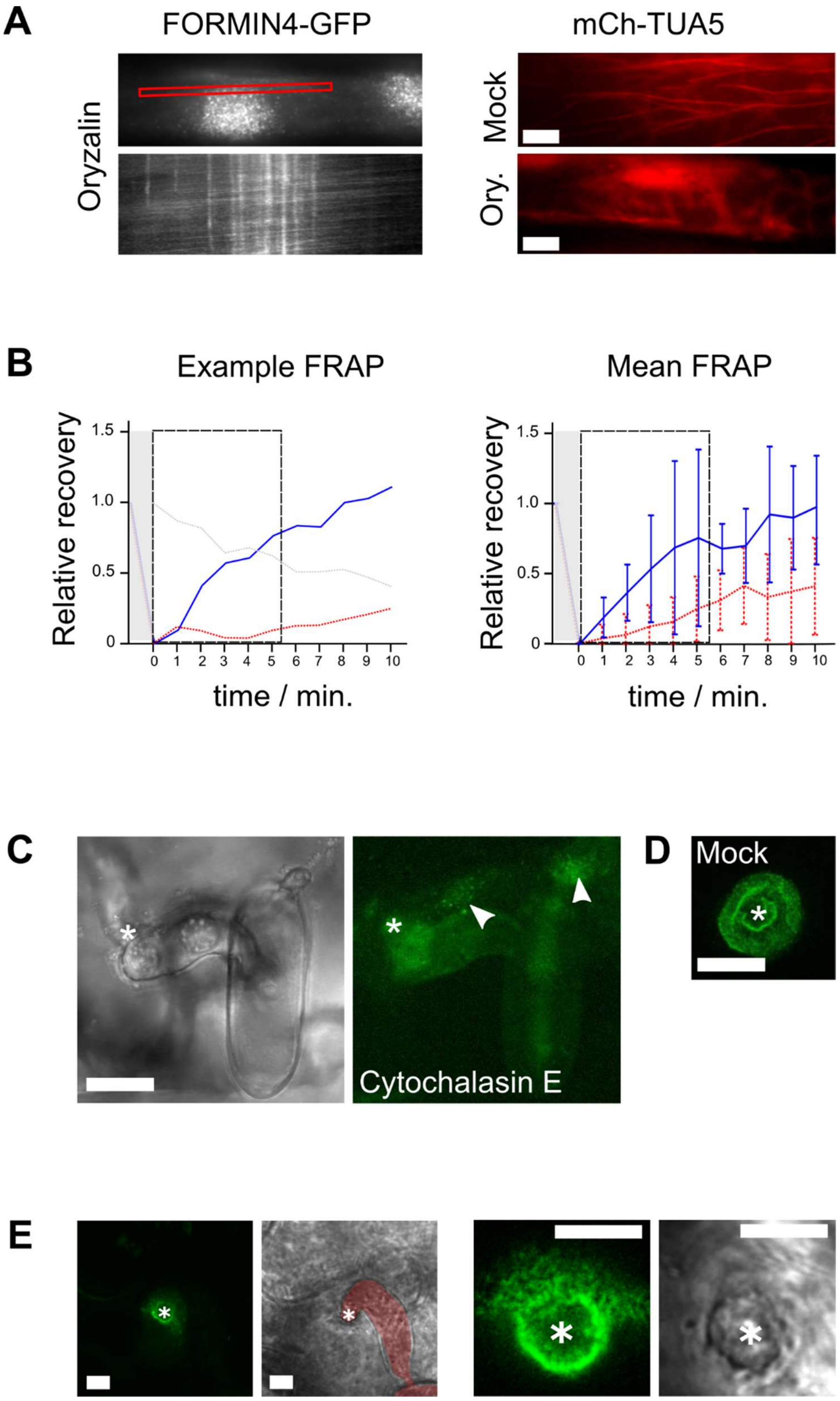

Figure S2. FORMIN4 transport and delivery requires actin but not its endogenous FH1-FH2 domain, Related to Figure 2.

(A) Oryzalin does not disrupt localisation or streaming of FORMIN4-GFP (white). Disruption of microtubules labelled using mCherry-TUA5 (red) confirmed the effectiveness of the oryzalin treatment. Red box is the source of the kymograph in the second panel and is 28.5 µm. Scale bar is 5 µm.

(B) Recovery plots of FRAP data shown in Figure 2. The left-hand plot shows an example recovery of a single FORMIN4-GFP region in an elicitor only (blue line) and latrunculin B (red line) treated hypocotyl. Grey dashed line indicates the reduction of signal in the whole field of view throughout the observation period. Bleach corrections were therefore applied to each data series using control FORMIN4-GFP regions (please see materials and methods). The right-hand plot shows the mean recovery of elicitor only (blue line) and latrunculin B (red line) treated hypocotyls. Black dashed rectangle indicates evaluated time frame for calculating the rates of initial recovery. Error bars are standard deviation.

(C) Infiltration of actin filament capping drug cytochalasin-E prior to *Bgh* infection prevents FORMIN4-GFP accumulation at CWAs. White arrowheads indicate residual responses to fungal contact. Asterisk denotes CWA. Scale bar is 10 µm.

(D) Mock treatments using equivalent concentrations of DMSO do not compromise FORMIN4-GFP delivery. Asterisk denotes CWA.

(E) Two laser scanning confocal microscopy images of *formin4/7/8* expressing FORMIN4-GFP and infected with *Bgh* showing localisation and punctate distribution equivalent to Col-0 genetic background (see Figure 1). Scale bars are 5 µm.

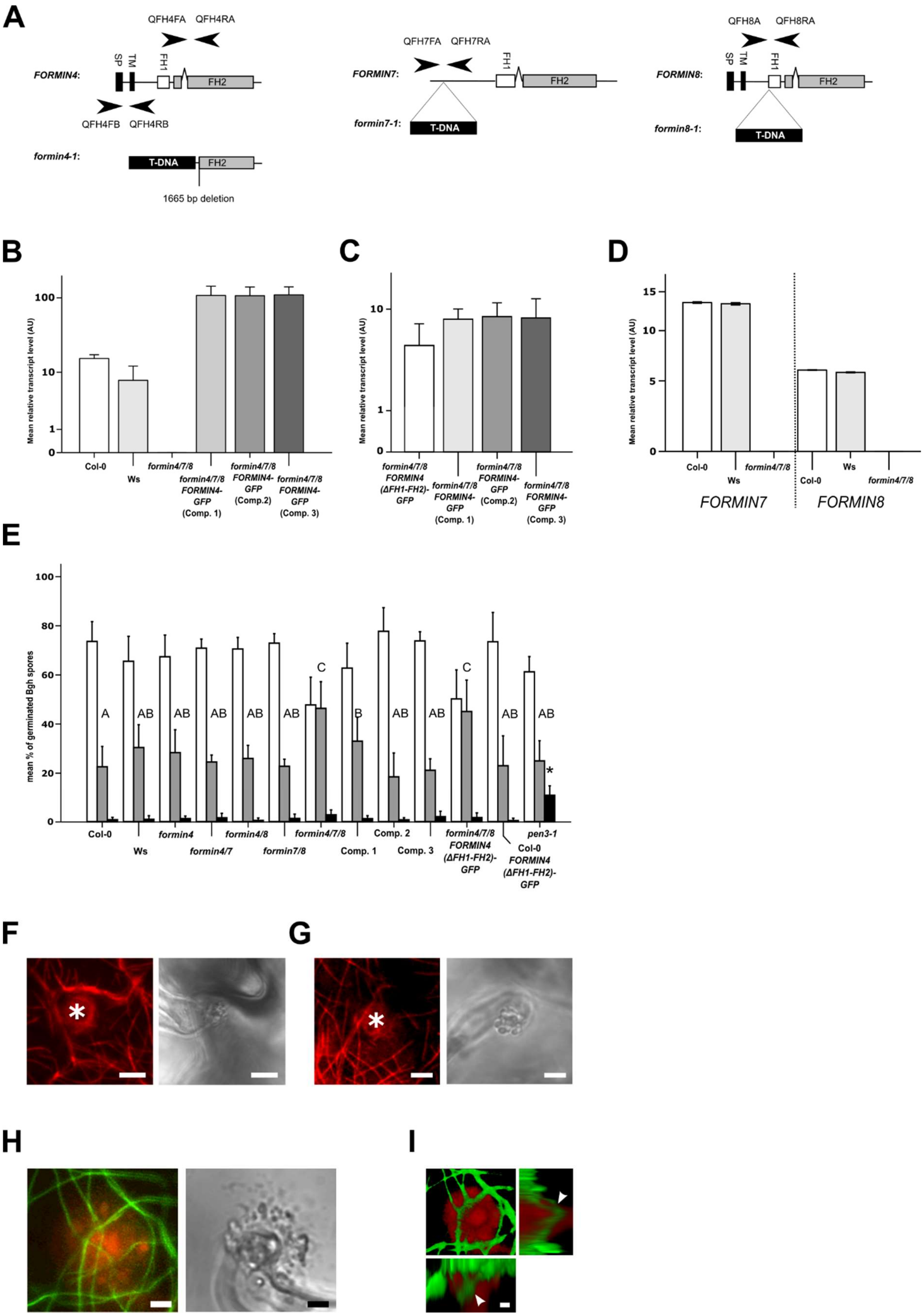

A
QFH4FA QFH4RA
FORMIN4:
SP TM FH1 FH2
QFH4FB QFH4RB
formin4-1:
T-DNA FH2
1665 bp deletion
QFH7FA QFH7RA
FORMIN7:
FH1 FH2
formin7-1:
T-DNA
QFH8A QFH8RA
FORMIN8:
SP TM FH1 FH2
formin8-1:
T-DNA
B
Mean relative transcript level (AU)
Col-0
Ws
formin4/7/8
formin4/7/8 FORMIN4-GFP (Comp. 1)
formin4/7/8 FORMIN4-GFP (Comp.2)
formin4/7/8 FORMIN4-GFP (Comp. 3)
C
formin4/7/8 FORMIN4 (ΔFH1-FH2)-GFP
D
FORMIN7
FORMIN8
E
mean % of germinated Bgh spores
A
AB
B
C
formin4
formin4/7
formin4/8
formin7/8
Comp. 1
Comp. 2
Comp. 3
Col-0 FORMIN4 (ΔFH1-FH2)-GFP
pen3-1
F
G
H
I

Figure S3. FORMIN4 contributes to defence against *Bgh*, Related to Figure 3 and Video S2.

(A) Diagram showing the relative positions of T-DNA insertions into mutant group 1e formin alleles. The arrows show the relative position of primers used for RT-QPCR analysis.

(B) Relative expression levels of *FORMIN4* in wild type, mutant and complemented mutant genotypes using primer pair QFH4FA/QFH4RA and control primer pair PTB1F/PTB1R (to housekeeping gene *ptb1*).

(C) Relative expression levels of *FORMIN4* in complemented mutant genotypes using terminal 5-prime primer pair QFH4FB/QFH4RB and control primer pair PTB1F/PTB1R.

(D) Relative expression levels of *FORMIN7 and FORMIN8* in wild type and triple mutant genotypes using primer pairs QFH7FA/QFH7RA and QFH8FA/QFH8RA respectively, in combination with control primer pair EF1F/EF1R (to elongation factor one alpha).

(E) Comparison of the frequency of defence response outcomes in different genotypes 48 hours after *Bgh* infection. These data are from additional biological repeats to those presented in Figure 3. Genotype 'comp.1' is equivalent to the complemented line quantified in Figure 3D. White bars = successful defence/papilla formation, grey bars = response resulted in cell death, black bars = successful haustoria formation. Significant groups were evaluated and defined by a generalized linear mixed model using Tukey HSD as post hoc test. Letters ('A' to 'C') denote groups of no significant difference ($p>0.05$) in proportion of cell death (relative to wild type and triple mutant genotypes). Compared to all other genetic backgrounds *formin4/7/8* and *formin4/7/8 FORMIN4(ΔFH1-FH2)-GFP* plants show increased rates of cell death (group C). Single asterisks indicates a significant difference in haustoria formation of the *pen3-1* mutant to all other tested lines (p-value < 0.001). Of the 129 haustoria recorded 20.2% were alive with no apparent significant difference between genotypes. Error bars show standard deviation of at least three biological repeats with a minimum of 4 leaves (reaching a total of 7119 penetration attempts).

(F) Col-0 leaf epidermal cell 48 hours after *Bgh* infection expressing mCherry-TUA5.

(G) Mutant *formin4/7//8* under the same conditions. Neither wild type nor mutant show microtubule bundling at CWAs. Scale bar is 5 µm.

(H) Maximum projection and accompanying bright field image of a laser scanning confocal microscope image stack of microtubule binding MAP4-GFP (green) and CWA autofluorecence (red) in a wild type genetic background showing microtubules in the zone of the CWA. Arrows indicate a microtubule bundle navigating the invaginated membrane around the CWA. Also see Video S2.

(I) 3D volume rendered image and orthogonal views of the *Bgh* interaction site. Arrowheads indicate microtubules (green) underlying/warping around the papilla (red). Scale bars are 2 µm.

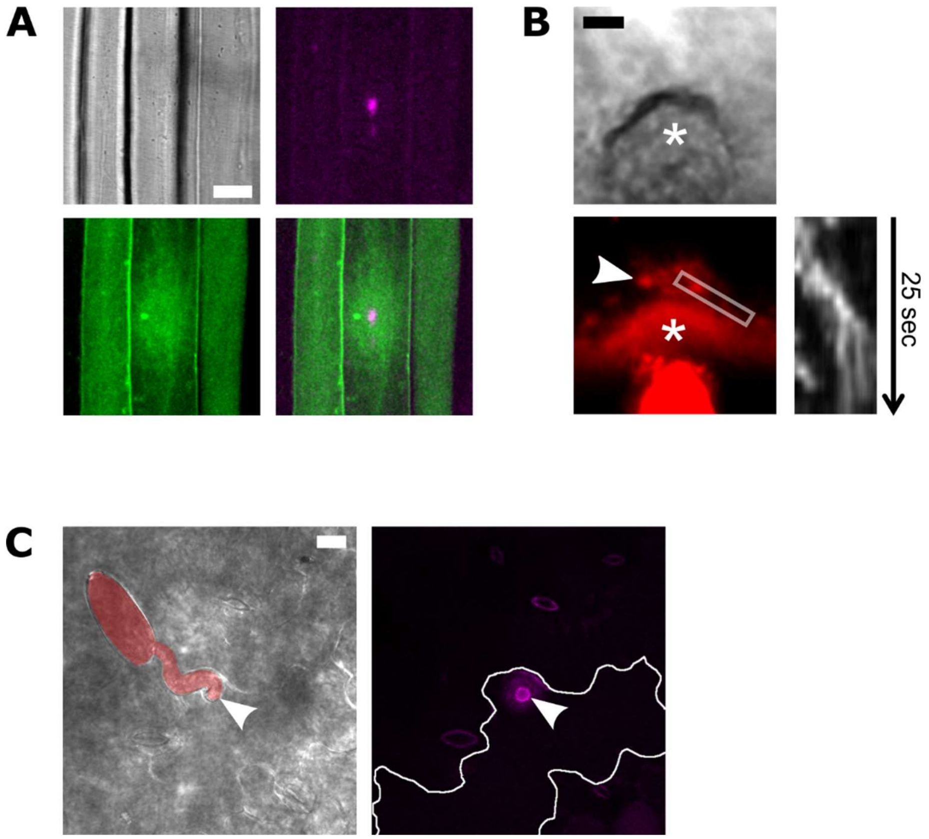

Figure S4. FORMIN4 transport and meso-localisation is distinct from PEN3, Related to Figure 4.

(A) PEN3-GFP (green channel) complementing *pen3-1* dark grown hypocotyls exposed to the elicitation treatment shown in Figure 2. Magenta channel shows aniline blue staining. Scale bar is 10 µm.

(B) The endosomal network (EN; indicated by white arrowhead) surrounding CWAs (example indicated by asterisk) was visualised through the uptake of FM 4-64 (red). Kymograph of the boxed area shows the movement and pausing behaviour of EN compartments. Scale bar is 2 µm.

(C) Tissue infected with *Bgh* expressing FORMIN4-tdTomato. Arrowheads indicate the CWA. Scale bar is 10 µm.

Table S1. Primer combinations for testing of homozygous T-DNA insertion lines.

Related to STAR Methods – Plant material.

**Allele**	**Primer 1**	**Primer 2**
FORMIN4 wild type	FH4GENFA	FH4GENRA
formin4-1 insert	FH4GENRB	TAG3
FORMIN7 wild type	FH7GENFA	FH7GENRA
formin7-1 insert	FH7GENFA	LB3
FORMIN8 wild type	FH8GENFA	FH8GENRA
formin8-1 insert	FH8GENFA	LB3
